# 2023 Brazilian Society of Rheumatology guidelines for the treatment of systemic sclerosis

**DOI:** 10.1186/s42358-024-00392-w

**Published:** 2024-07-10

**Authors:** Cristiane Kayser, Sandra Maximiano de Oliveira Delgado, Adriana Fontes Zimmermann, Alex Magno Coelho Horimoto, Ana Paula Toledo Del Rio, Carolina de Souza Müller, Cintia Zumstein Camargo, Cristiano Michelini Lupo, Daniela Aparecida de Moraes, Eduardo José Do Rosário E Souza, Flávia Patrícia Sena Teixeira Santos, Juliana Yuri Sekiyama, Lilian Scussel Lonzetti, Lucas Victória de Oliveira Martins, Mailze Campos Bezerra, Markus Bredemeier, Maria Carolina Oliveira, Maria Cecília da Fonseca Salgado, Renata Miossi, Sheila Márcia de Araújo Fontenele, Vanessa Hax, Andrea Tavares Dantas, Percival Degrava Sampaio-Barros

**Affiliations:** 1 Rheumatology Division, Escola Paulista de Medicina, Universidade Federal de São Paulo—UNIFESP, Rua dos Otonis 863, 2º andar, Vila Clementino, São Paulo, SP 04025-002 Brazil; 2 Rheumatology Division, Hospital Sírio Libanês, Brasília, DF Brazil; 3 Rheumatology Division, Professor Polydoro Ernani de São Tiago University Hospital, Universidade Federal de Santa Catarina—UFSC, Florianópolis, SC Brazil; 4 Rheumatology Division, Hospital Regional do Mato Grosso do Sul, Faculdade de Medicina, Universidade Federal de Mato Grosso do Sul—UFMS, Campo Grande, MS Brazil; 5 Rheumatology Division, Universidade Estadual de Campinas—UNICAMP, Campinas, SP Brazil; 6 Rheumatology Division, Hospital de Clínicas, Universidade Federal do Paraná—UFPR, Curitiba, PR Brazil; 7 Internal Medicine Departament, Faculdade de Medicina de Botucatu, Universidade Estadual Paulista—UNESP, Botucatu, SP Brazil; 8 Rheumatology Division, Faculdade de Medicina de São José do Rio Preto—FAMERP, São José do Rio Preto, SP Brazil; 9 Medicine School, Centro Universitário Barão de Mauá—CBM, Ribeirão Preto, SP Brazil; 10 Rheumatology Division, Hospital de Santa Casa de Belo Horizonte, Belo Horizonte, MG Brazil; 11 Rheumatology Division, Universidade Federal de Minas Gerais—UFMG, Belo Horizonte, MG Brazil; 12 Internal Medicine Departament, Universidade Estadual de Maringá—UEM, Maringá, PR Brazil; 13 Rheumatology Division, Universidade Federal de Ciências da Saúde de Porto Alegre—UFCSPA, Porto Alegre, RS Brazil; 14 Rheumatology Division, Universidade Federal do Ceará—UFC, Fortaleza, CE Brazil; 15 Rheumatology Division, Hospital Nossa Senhora da Conceição, Porto Alegre, RS Brazil; 16 Clinical Immunology Division, Ribeirão Preto Medical School, Universidade de São Paulo—USP, Ribeirão Preto, SP Brazil; 17 Rheumatology Division, Universidade Federal do Estado do Rio de Janeiro—UNIRIO, Rio de Janeiro, RJ Brazil; 18 Rheumatology Division, Hospital das Clínicas HCFMUSP, Faculdade de Medicina, Universidade de São Paulo—USP, São Paulo, SP Brazil; 19 Rheumatology Division, Universidade Estadual do Ceará—UECE, Fortaleza, CE Brazil; 20 Rheumatology Division, Hospital de Clínicas de Porto Alegre, Universidade Federal do Rio Grande do Sul—UFRGS, Porto Alegre, RS Brazil; 21 Rheumatology Division, Universidade Federal de Pernambuco—UFPE, Recife, PE Brazil

**Keywords:** Systemic sclerosis, Scleroderma, Treatment, Disease management, Interstitial lung disease, Raynaud’s phenomenon, Digital ulcer, Guidelines

## Abstract

**Background:**

Systemic sclerosis (SSc) is a rare chronic autoimmune disease with heterogeneous manifestations. In the last decade, several clinical trials have been conducted to evaluate new treatment options for SSc. The purpose of this work is to update the recommendations of the Brazilian Society of Rheumatology in light of the new evidence available for the pharmacological management of SSc.

**Methods:**

A systematic review including randomized clinical trials (RCTs) for predefined questions that were elaborated according to the Patient/Population, Intervention, Comparison, and Outcomes (PICO) strategy was conducted. The rating of the available evidence was performed according to the Grading of Recommendations Assessment, Development and Evaluation (GRADE) methodology. To become a recommendation, at least 75% agreement of the voting panel was needed.

**Results:**

Six recommendations were elaborated regarding the pharmacological treatment of Raynaud’s phenomenon, the treatment (healing) and prevention of digital ulcers, skin involvement, interstitial lung disease (ILD) and gastrointestinal involvement in SSc patients based on results available from RCTs. New drugs, such as rituximab, were included as therapeutic options for skin involvement, and rituximab, tocilizumab and nintedanib were included as therapeutic options for ILD. Recommendations for the pharmacological treatment of scleroderma renal crisis and musculoskeletal involvement were elaborated based on the expert opinion of the voting panel, as no placebo-controlled RCTs were found.

**Conclusion:**

These guidelines updated and incorporated new treatment options for the management of SSc based on evidence from the literature and expert opinion regarding SSc, providing support for decision-making in clinical practice.

**Supplementary Information:**

The online version contains supplementary material available at 10.1186/s42358-024-00392-w.

## Background

Systemic sclerosis (SSc) is a rare systemic autoimmune disease characterized by a triad of microvascular damage, innate and adaptive immune dysregulation, and progressive tissue fibrosis of the skin and internal organs [1]. SSc is a heterogeneous disease with an unpredictable clinical course and high morbidity and mortality rates [2]. Different organs and systems can be affected, including the skin, gastrointestinal tract, blood vessels, musculoskeletal system, heart, lungs, and kidneys [3].

Patients with SSc can be classified into two major clinical subtypes with distinct rates of disease progression, according to the extent of skin involvement. Patients with diffuse cutaneous SSc (dcSSc) have a rapid progression of skin thickening, localized proximal to the elbows or knees, a higher risk of visceral involvement and a higher mortality rate [1]. On the other hand, in patients with limited cutaneous SSc (lcSSc), the skin thickening is restricted to areas distal to the elbows and knees, with or without involvement of the face. In those patients, visceral involvement is generally less frequent, but there is a higher risk of late-stage development of pulmonary arterial hypertension (PAH) [1]. Those patients without skin thickening but presenting visceral and vascular manifestations of SSc with positive antinuclear antibodies (ANA), were considered *SSc sine scleroderma* [4]. Aiming at an early diagnosis of the disease, patients might also be classified as having “early” or “very early” SSc [5, 6].

Treatment of SSc can be challenging, particularly due to the heterogeneous disease manifestation and considerable variability in the treatment response [7]. Nonetheless, in the last decade, several randomized controlled trials (RCTs) have been performed, and advances in the management of SSc have been made [7]. Moreover, a better comprehension of the disease pathogenesis and the clinical course of SSc has reinforced the importance of early diagnosis and early detection of organ involvement [8], which might impact patient prognosis. Thus, current treatments should be stratified according to organ involvement based on early screening and careful evaluation of each patient.

The aim of these guidelines is to provide new recommendations for the pharmacological treatment of SSc, except for PAH, for which specific guidelines have recently been published [9–11]. This version replaces the previous guidelines published in 2013 [12] and is grouped based on organ involvement, reflecting decision-making in clinical practice. It is also important to point out that the recommendations made in this paper do not intend to replace the patient‒physician shared decision or personalized treatment based on the rheumatologist’s evaluation and are intended to provide updated scientific evidence to help clinicians treat their patients in clinical practice.

## Methods

The elaboration of these guidelines was conducted according to the Grading of Recommendations Assessment, Development and Evaluation (GRADE) methodology to assess the quality of the evidence and the strength of the recommendation [13], mainly regarding the balance between benefits and harms, values and preferences, costs, certainty of evidence and equity. Recommendations could be either in favor of or against the proposed intervention and either strong or weak/conditional. Thus, the recommendations were classified into four categories: *strongly recommended, conditionally recommended, conditionally recommended against, and strongly recommended against* a given intervention [13]. It was an initiative of the Systemic Sclerosis Committee from the Brazilian Society of Rheumatology (SBR), a committee composed of rheumatologists with interest and experience in SSc.

First, eleven clinical questions regarding SSc treatment were elaborated by a core leadership team consisting of four rheumatologists with expertise in SSc (C.K., S.M.O.D., A.T.D., and P.D.S.B.) according to the Patient/Population, Intervention, Comparison, Outcome (PICO) strategy. The clinical questions were validated in a virtual meeting by a panel of experts, consisting of 23 rheumatologists of the SBR SSc Committee. The PICO question regarding physical rehabilitation for musculoskeletal involvement in SSc patients was discarded by the expert panel because this work aimed to review pharmacological interventions. The PICO question about PAH was later discarded based on the presence of specific guidelines recently published [9–11]. The PICO question about cardiac involvement was discarded by the expert panel due to the lack of scientific evidence for its management. As no placebo-controlled clinical trial for the treatment of scleroderma renal crisis (SRC) and musculoskeletal involvement was found and due to the importance of these two clinical questions, the authors elaborated these two recommendations based on case‒controlled or head-to-head studies and on the expert opinion of the voting panel. The core leadership team also prespecified the outcomes that were considered critical or important for each PICO question for the systematic literature review.

According to the GRADE methodology, a strong recommendation is one for which the expert panel is confident that the desirable effects of an intervention outweigh its undesirable effects (strong recommendation for an intervention) or that the undesirable effects of an intervention outweigh its desirable effects (strong recommendation against an intervention) [13]. A weak or conditional recommendation is one for which the desirable effects probably outweigh the undesirable effects or undesirable effects probably outweigh the desirable effect, but appreciable uncertainty exists (Table 1). Moreover, the quality of evidence was categorized into four categories: high, moderate, low, or very low (Table 2). Agreement between panelists was determined by the Delphi technique [14] using an online anonymous survey, and a minimum of 75% agreement was needed for each recommendation. The methodological details, including PICO questions, outcomes and the search strategy for the systematic review, are available in the Supplementary material (Supplementary Chart 1 and Supplementary Tables 1 and 2). The entire process was supervised by two expert methodologists (A.R. and V.T.C.) from the SBR research unit.

**Table 1:** Strength of the recommendation according to GRADE

Strength of the recommendation	Description
Strong recommendation	The desirable effects of intervention clearly outweigh its undesirable effects
Conditional recommendation	The desirable effects probably outweigh the undesirable effects, but appreciable uncertainty exists
Conditional recommendation against	The undesirable effects probably outweigh the desirable effect, but appreciable uncertainty exists
Strong recommendation against	The undesirable effects of an intervention outweigh its desirable effect

**Table 2:** GRADE Working Group grades of certainty

Certainty	
High	The authors have a lot of confidence that the true effect lies close to the estimated effect
Moderate	The authors are moderately confident that the true effect is probably close to the estimated effect
Low	The confidence in the estimated effect is limited: the true effect may be substantially different from the estimated effect
Very low	The authors have very little confidence in the estimated effect: the true effect is likely to be substantially different from the estimated effect

### Search strategy and study selection

A systematic review was performed in accordance with the Preferred Reporting Items for Systematic Reviews and Meta-Analyses (PRISMA) guidelines [15] to elaborate recommendations for the pharmacological treatment of systemic sclerosis.

A systematic literature search including RCTs in adult patients with SSc was performed. Electronic searches were performed in MEDLINE (via PubMed) (from 1966 until May 26th, 2021), Embase (via Elsevier) (from 1974 until May 26th, 2021), Lilacs (via Portal Regional da Biblioteca Virtual de Saúde [BVS]) (from 1982 until May 26th, 2021), and Cochrane Central Register of Controlled Trials (CENTRAL)/Cochrane Library (5th Edition, 2021), without language limitations, using Medical Subject Heading (MeSH) terms for all clinical questions (Supplementary Table 3).

Rayyan *software* [16] was used for the literature screening process. Two independent reviewers (A.R. and V.T.C.) screened titles and abstracts that were potentially eligible according to the following inclusion criteria: patients older than 18 years of age with SSc and RCTs. Next, all articles underwent a full-text review by the same two independent reviewers aiming for definite inclusion in the meta-analysis. Disagreements were resolved by a third reviewer (A.C.P.). Studies judged by the expert panel as interventions already considered to be out of date (e.g., penicillamine, relaxin, and lidocaine) and head-to-head comparisons without previous RCTs against placebo were excluded. Interventions with ambrisentan, riociguat, belimumab, imatinib, and lenabasum were excluded from the analysis due to the lack of clinical benefit on RCT. Additionally, a further analysis of additional studies considered relevant that were missed by the screening process was performed by the core leadership team. Two additional clinical studies [17, 18] that fulfilled the inclusion criteria and were published between May 2021 and July 2022 were included in this review by the decision of the core leadership team.

### Statistical analysis

For continuous data, the mean, standard deviation (SD) and number of participants in each intervention group of the included trials were extracted. If the data were presented in other measurements (for example, confidence interval [CI] or standard error [SE]), the SD was calculated as previously described [19]. Data were summarized using the mean difference (MD) or standardized mean difference (SMD) and 95% CI by means of meta-analyses by generic inverse variance or the inverse variance method and the random effects model. Dichotomous outcomes were expressed using the risk ratio (RR) in random effects meta-analyses (with the inverse variance method or generic inverse variance). All data were analyzed using Review Manager 5 software.

Outcome data were extracted, when available, based on an intention-to-treat (ITT) analysis (with all participants randomized) or a modified ITT analysis (with assumptions decided upon by the study authors). If necessary, the principal author of each included study was contacted to obtain any missing study characteristics or outcome data. If the data were not available or the study authors did not respond, only the analysis with the available data was performed. In this case, the impact of including these trials on the overall assessment of the meta-analyses was explored by a sensitivity analysis. In the case of continuous data, the SMD was calculated based on the number of participants analyzed at the last follow-up. Search strategy details, assessment of quality, and risk of bias assessment are shown in the Supplementary material.

## Results and recommendations

**Fig. 1:**
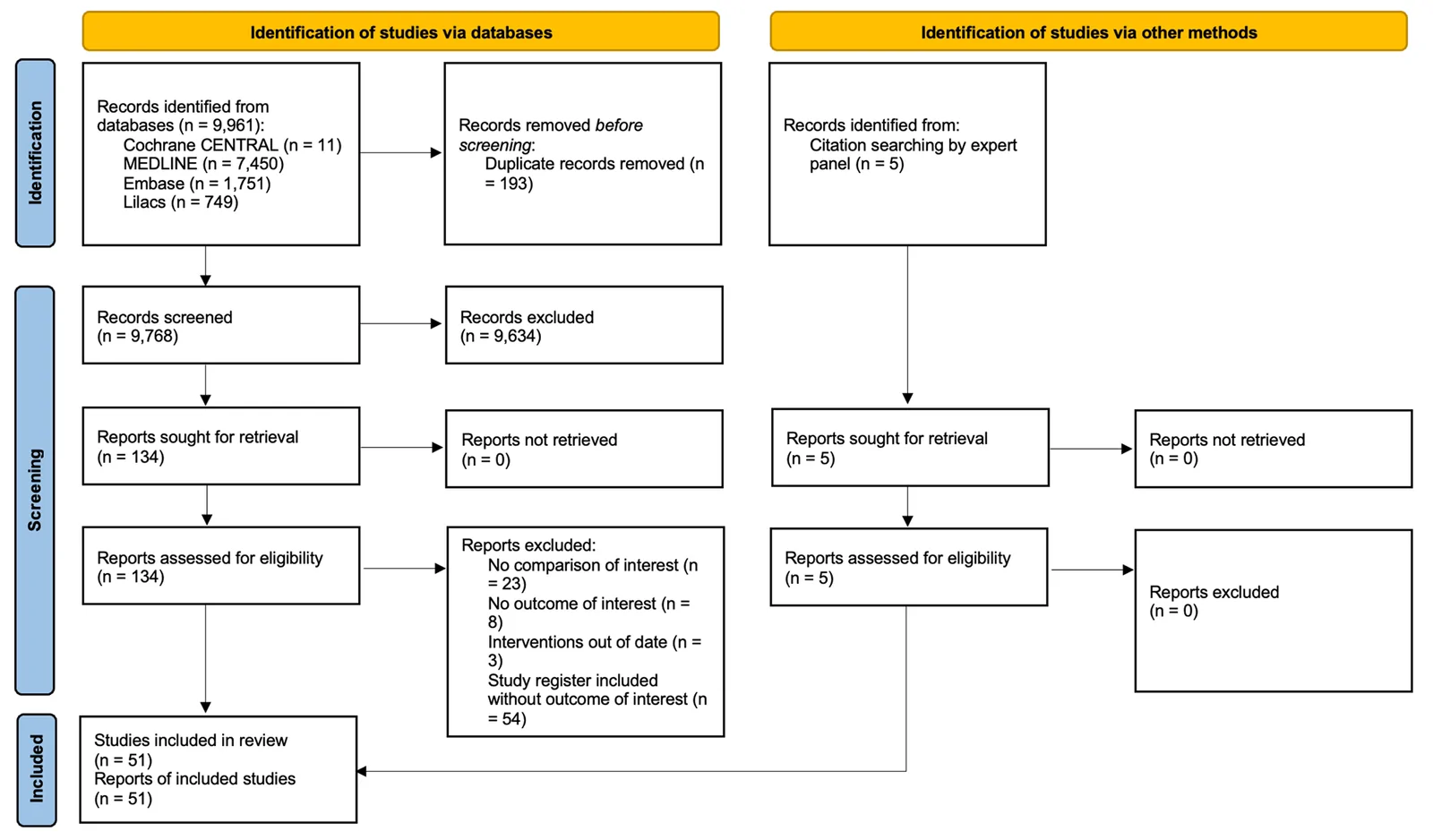
PRISMA flowchart of the selection and eligibility of the studies

The search retrieved 9961 records from the databases (7450 in MEDLINE, 1751 in Embase, 749 in Lilacs, and 11 in CENTRAL). After eliminating duplicates, 9768 references were evaluated by reading titles and abstracts. Of these, 9634 records were excluded. Thus, 134 full-text articles were considered for the evidence report and assessed for eligibility. After reviewing the full-text articles, 88 studies were excluded for several reasons (Fig. 1). Five additional records were also identified by the core leadership team and included manually [17, 18, 20–22]. Thus, 51 clinical trials were included in this review. Full details regarding the selection stage and inclusion of studies are presented in Fig. 1.

**Fig. 2:**
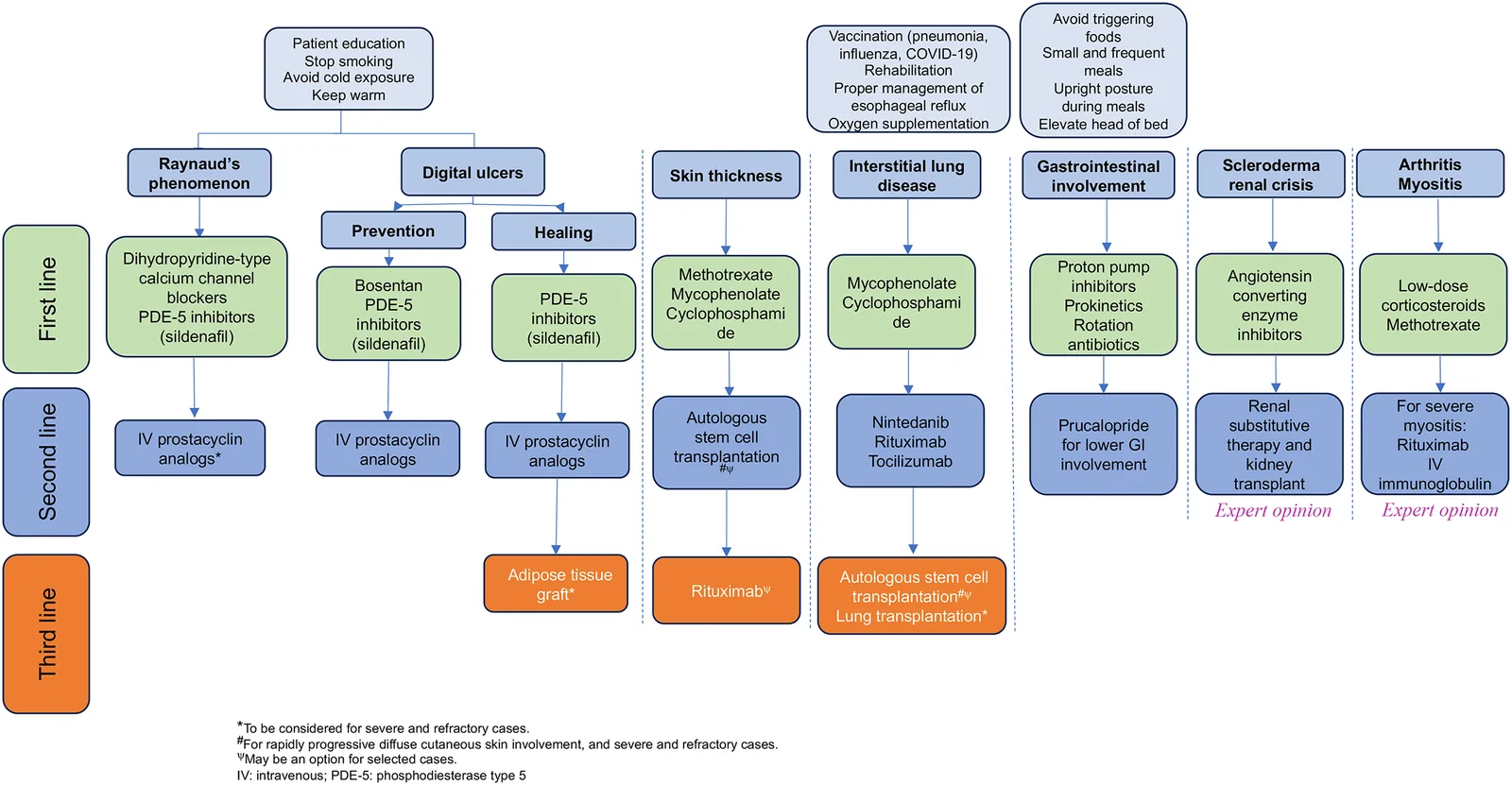
Brazilian Rheumatology Society flowchart of systemic sclerosis treatment

A summary of the recommendations, certainty of evidence and degree of agreement for each elaborated clinical question is presented in Table 3. The following recommendations for the management of SSc are summarized in Fig. 2.

**Table 3:** Recommendations of the Brazilian Society of Rheumatology for the pharmacological management of patients with systemic sclerosis

Clinical question	Recommendation	Strength of recommendation	Quality of evidence	Degree of agreement (%)
What is the evidence for the pharmacological treatment of Raynaud’s phenomenon?	Calcium channel blockers (dihydropyridine-type)	Strong	Very low	87
	PDE-5 inhibitors (sildenafil)	Strong	Moderate	95.7
	Intravenous prostacyclin analogs	Conditional	Very low	91.3
What evidence is available for the treatment of digital ulcers (DUs)?	Phosphodiesterase type 5 inhibitors	Strong	Low	100
	Intravenous prostacyclin analogs	Conditional	Very low	95.7
	Adipose tissue graft	Conditional	Very low	95.7
What evidence is available for preventing recurrence of DUs?	Bosentan	Strong	Moderate	82.6
	Phosphodiesterase type 5 inhibitors	Strong	Low	100
	Intravenous prostacyclin analogs	Conditional	Very low	95.7
What is the evidence for the treatment of skin involvement?	Methotrexate	Strong	Moderate	96
	Mycophenolate	Conditional	Low	83
	Cyclophosphamide	Conditional	Moderate	87
	Rituximab	Conditional	Low	96
	Autologous stem-cell transplantation	Strong	Moderate	78.3
What is the evidence for the treatment of interstitial lung disease?	Cyclophosphamide	Conditional	Low	78
	Mycophenolate	Conditional	Low	91
	Nintedanib	Conditional	Low	87
	Rituximab	Conditional	Low	83
	Tocilizumab	Conditional	Moderate	91
	Autologous stem-cell transplantation	Conditional	Low	96
What therapeutic evidence is available for the treatment of gastrointestinal involvement?	Prucalopride	Conditional	Very low	95.7
What treatments are beneficial in the management of scleroderma renal crisis?	Angiotensin-converting enzyme inhibitors	–	Expert opinion	87
What treatments are beneficial in the management of musculoskeletal manifestations (arthritis and/or myositis)?	Low-dose corticosteroids and methotrexate	–	Expert opinion	78.3

*DU* digital ulcer, *PDE-5* phosphodiesterase type 5

### What is the evidence for the pharmacological treatment of Raynaud’s phenomenon (RP) in patients with SSc?

RP is the most common manifestation of SSc, affecting approximately 95% of patients. RP is typically the first clinical manifestation of SSc, and it can precede the diagnosis of SSc by many years, especially in patients with lcSSc [23]. Recent studies have shown that RP can have a major impact on the quality of life of SSc patients [24]. RP treatment includes patient education and lifestyle measures, including avoiding cold exposure and stopping smoking [23, 24].

Four RCTs evaluated the efficacy of nifedipine, a calcium channel blocker (CCB), 10–20 mg three times daily compared to placebo for one to six weeks in the treatment of RP in SSc patients [25–28]. A total of 50 patients were evaluated. There was a clinically meaningful reduction in the frequency of RP attacks/week (MD  −5.5, 95% CI  −11.5 to 0.4) and in the duration of RP attacks (MD  −13.4 min, 95% CI  −40.4 to 13.7) and a significant reduction in the severity of RP attacks in the Visual Analog Scale (VAS) (0–10 cm) (MD  −2.4 cm, 95% CI  −4.1 to −0.7) with nifedipine compared to placebo. Although the evidence is of very low quality, the expert panel strongly recommended dihydropyridine-type CCBs, especially nifedipine, for the treatment of SSc-RP. Given their availability and safety profile, CCBs should be the first treatment choice in these patients.

Eight RCTs evaluated the efficacy of phosphodiesterase type 5 (PDE-5) inhibitors, four with sildenafil, three with tadalafil, and one with vardenafil, compared to placebo for the treatment of RP in SSc [22, 29–35]. A total of 348 patients were included. PDE-5 inhibitors led to a significant reduction in the frequency of attacks (MD  −0.74 attacks/day, 95% CI  −1.37 to  −0.12) and in the duration of RP attacks (MD  −15.2 min, 95% CI −23.4 to −6.6) compared to placebo. There was also an MD reduction of −0.48 (95% CI −1.79 to +0.77) in the severity of attacks evaluated using Raynaud’s Condition Score (RCS) (scale of 0–10). Sildenafil is the most commonly available drug and has a higher number of RCTs. Sildenafil was first evaluated by Fries et al. in 2005 in a double-blinded, crossover study in 16 patients with symptomatic RP at a dose of 50 mg twice daily [32]. The use of sildenafil was associated with a significant reduction in the mean frequency and duration of RP attacks, as well as in the RCS compared to placebo [32]. In the study by Andrigueti et al., there was a significant reduction in the duration of RP after 8 weeks of sildenafil 20 mg three times daily compared with placebo (mean % change of −39.1% versus −1.2%; *p* = 0.042) [30]. In the SEDUCE study, an RCT aiming at evaluating the efficacy of sildenafil on digital ulcer healing, no significant difference was observed in RP severity between groups [35]. Finally, an RCT evaluated the use of a modified-release sildenafil, 200 mg once a day for 25 days versus placebo. A significant reduction in the percentage of attacks per week in the sildenafil group compared to placebo was found (−44% versus −18.1%, *p* = 0.034) [33]. Based on these results, the expert panel strongly recommended PDE-5 inhibitors, especially sildenafil, as first-line treatment for the treatment of SSc-RP.

Intravenous (IV) prostacyclin analogs, particularly iloprost, were evaluated in three RCTs compared to placebo for the treatment of SSc-RP [36–38]. Oral iloprost was also evaluated in three RCTs [39–41]. IV iloprost (0.5–2.0 ng/kg/min) for 3–5 consecutive days reduced the frequency and duration of RP attacks (MD −0.66 attacks/day, 95% CI −1.47 to −0.15, and −38 min, 95% CI −77 to 1, respectively) but not the severity of the attacks evaluated by the RCS (MD 0.29, 95% CI −0.52 to 1.09). Oral iloprost led to a smaller decrease in the frequency and duration of RP attacks compared to placebo (MD −0.48 attacks/day, 95% CI −1.24 to 0.28; and −34 min, 95% CI −132 to 64, respectively) and to a significant decrease of −0.40 (95% CI −0.77 to −0.22) in the severity of attacks. When evaluated together, IV and oral iloprost led to a small reduction in the frequency and severity of RP attacks (MD −0.9 attacks per day, 95% CI −1.64 to −0.15; and −0.26 cm, 95% CI −0.82 to 0.29, respectively), as well as in the duration of RP attacks (−26 min, 95% CI −60 to 7). Although available in Brazil, inhaled iloprost was not considered, as no RCT has evaluated its role in the treatment of RP. Considering cost, availability, and the very low quality of evidence, IV prostacyclin analogs were conditionally recommended for the treatment of SSc-RP. As oral or IV iloprost is not available in our country, the panelists suggested that alprostadil (60 mcg/day for 5–7 days), another IV prostacyclin analog with significantly lower cost, could be used for the treatment of refractory and severe cases [42].

Prazosin, an alpha1-adrenergic blocker, was evaluated in one RCT with 21 SSc patients [26]. After one week of prazosin 1 mg three times daily or placebo, prazosin did not show a significant decrease in the frequency of RP attacks/week or in the severity of the attacks (VAS). Thus, the expert panel conditionally recommended against treatment with this drug.

Five other treatments evaluated in RCTs, including atorvastatin [43], bosentan [44], selexipag [20], botulinum toxin [21], and adipose tissue-derived stromal vascular fraction [18], were strongly recommended against its use due to lack of clinical efficacy and/or very low quality of the studies. Although increasing interest in botulin toxin injections exists, at the present time, the results of one RCT did not show a clear benefit for its use, with only a small decrease in the severity of RP, and further RCTs are needed. Details regarding these studies, including outcomes, evaluation instruments, measures of effect, certainty of the evidence, recommendations, and agreement, are described in Supplementary Table 4.

#### Recommendation 1

Calcium channel blockers, especially dihydropyridine-type, and PDE-5 inhibitors, mainly sildenafil, are strongly recommended for the treatment of RP. Intravenous prostacyclin analogs are conditionally recommended for the treatment of severe or refractory cases.

### What evidence is available for the treatment of digital ulcers (DUs) in patients with SSc?

Digital vasculopathy occurs in virtually all SSc patients, and nearly 50% of them will develop a DU during the disease course [45]. Nearly 75% of patients who develop DUs will develop their first lesion within the first five years of the onset of the disease. The occurrence of DUs is associated with poor quality of life in such patients due to pain and hand disability and the risk of complications such as gangrene, infection and digital amputation [45, 46]. In addition, the presence of DU is related to a worse prognosis and a higher risk of internal organ involvement [46].

One RCT evaluated the efficacy of sildenafil 20 mg three times daily compared to placebo for 12 weeks in the treatment of DU [35]. A reduction in the number of DUs favored sildenafil against placebo (MD −0.6, 95% CI −1.56 to 0.36), showing an improvement in the healing rate of DUs in SSc. Based on these results, the expert panel strongly recommended the use of PDE-5 inhibitors, mainly sildenafil, as the first-line option for the treatment of DUs in SSc.

Iloprost was also evaluated in one RCT regarding its efficacy in healing DUs in SSc [37], and complete healing of cutaneous lesions was observed in the participants 10 weeks after treatment (RR 8.12, 95% CI 0.57–115.07). Additionally, two RCTs evaluated the efficacy of adipose tissue graft for healing DUs in SSc, suggesting improvement in hand function and in DU healing (RR 2.91, 95% CI 0.27–31.73) [18, 47]. Thus, the expert panel conditionally recommended the use of IV prostacyclin analogs as second-line therapy for the treatment of DUs. As iloprost is not available in our country, the panelists suggested that alprostadil, another IV prostacyclin analog, could be used for the treatment of refractory and severe cases. Adipose tissue graft should be considered for severe and refractory cases.

Further studies evaluated the efficacy of botulinic toxin injection [21], macitentan [48] and selexipag [20] for healing DUs in SSc. However, due to the lack of clinical efficacy and/or very low quality of the studies, we strongly recommended against the use of these therapeutic options. One study evaluated the efficacy of atorvastatin (40 mg/day for 4 months) in patients with SSc who experienced DU despite vasodilator therapy and demonstrated a decrease in the overall number of DU and the appearance of new DU, compared to placebo [49], suggesting atorvastatin as a potential adjuvant therapy for DU in SSc patients. The outcome, evaluation instruments, measures of effect, certainty of the evidence, recommendation and agreement of these studies are detailed in Supplementary Table 5.

#### Recommendation 2

PDE-5 inhibitors are strongly recommended as first-line options for the healing of DUs in SSc. IV prostacyclin analogs are conditionally recommended as a second-line option, while adipose tissue graft should be considered (conditionally recommended) for severe and refractory cases.

### What evidence is available for pharmacological management in the prevention of DU recurrence in patients with SSc?

Two RCTs evaluated the efficacy of bosentan, an endothelin receptor antagonist, for healing DUs and preventing their recurrence [50, 51]. Oral bosentan taken twice daily reduced the occurrence of new DUs in comparison with placebo (SMD −1.22 new DU/patient, 95% CI −1.58 to −0.87) but did not improve the healing of existing DUs [50, 51]. The quality of the evidence was considered moderate. Based on these results, the expert panel strongly recommended the use of bosentan for preventing DU recurrence in SSc patients.

The efficacy of PDE-5 inhibitors, mainly sildenafil, in reducing the number of new DUs in patients with SSc was also evaluated in one RCT, and a decrease in the number of DUs favoring sildenafil against placebo was shown (RR 0.55, 95% CI 0.26–1.14) [35]. Although the quality of evidence was low, due to their availability and safety profile, PDE-5 inhibitors were strongly recommended by the expert panel for preventing the recurrence of DUs.

One RCT evaluated the efficacy of iloprost in preventing DU recurrence in SSc [37], demonstrating a positive effect (RR 1.18, 95% CI 0.30–4.72). As the quality of evidence was very low and considering cost and availability, the panelists conditionally recommended the use of IV prostacyclin analogs for the prevention of DU recurrence in SSc.

Botulinic toxin injection [21] and macitentan [48] were also evaluated for preventing the recurrence of DUs in SSc. Nevertheless, due to the lack of clinical efficacy and/or very low quality of the studies, we strongly recommended against the use of these therapeutic options. Specific details of these studies are described in Supplementary Table 6.

#### Recommendation 3

Bosentan and PDE-5 inhibitors are strongly recommended for the prevention of DU recurrence in SSc. IV prostacyclin analogs are conditionally recommended for recurrent DUs in SSc.

### What is the evidence for the treatment of skin involvement in patients with SSc?

Skin fibrosis is considered the hallmark of SSc and allows the classification of patients into the two main clinical forms, dcSSc and lcSSc, according to the extent of this involvement. Less than 5% of patients have SSc *sine scleroderma* [52]. The progression of skin involvement is associated with a higher frequency of internal organ involvement and higher mortality in the disease [53]. In addition, the extent of cutaneous involvement, assessed by the modified Rodnan skin score (mRSS), is directly related to greater disability, especially hand disability, pain, fatigue and worse quality of life [54].

Two RCTs evaluated the effect of methotrexate (MTX), both oral and intramuscular, at doses of 10–25 mg/week for up to 12 months compared to placebo in 100 patients with early SSc [55, 56]. There was a decrease in the mRSS (MD −5.17 points, 95% CI −10.08 to −0.13), with the quality of evidence rated as moderate. Based on these results, the expert panel strongly recommended MTX for the treatment of skin involvement in SSc patients.

The efficacy of oral cyclophosphamide (CYC) (1–2 mg/kg for 12 months) versus placebo in the treatment of skin thickening was evaluated as a secondary endpoint in the Scleroderma Lung Study (SLS I), which included 145 patients with dcSSc or lcSSc [57] and showed a mRSS decrease of 2.7 points (MD −2.7 points, 95% CI −5.53 to 0.13). Another study compared the efficacy of oral CYC with azathioprine (AZA) in 60 patients with early dcSSc, showing a decrease in mRSS in favor of CYC (MD −9.67, 95% CI −9.98 to −9.36) [57]. Considering the quality of evidence, the safety profile of the medication, and availability, the expert panel considered a conditional recommendation of CYC in the treatment of skin fibrosis. Based on the result of the aforementioned RCT comparing AZA and oral CYC for early dcSSc [58] and on the low quality of the evidence, AZA was not recommended by the expert panel for the treatment of cutaneous involvement in SSc. Nonetheless, it could be considered a therapeutic option for maintenance treatment after induction treatment with other immunosuppressants for selected cases.

One RCT evaluated the efficacy of mycophenolate mofetil (MMF) (2 g/day) versus placebo in 41 SSc patients over six months, showing a 3.3-point reduction in the mRSS (95% CI −6.18 to −0.42) [59]. In the Scleroderma Lung Study II (SLS II), MMF (target dose: 1.5 g twice daily) was administered for 24 months in one arm, and oral CYC (target dose: 2 mg/kg/day) was administered for 12 months followed by placebo for 12 months in the other arm. The effect on the mRSS was assessed as a secondary endpoint, and no differences were demonstrated between the two treatment arms (MD 0.45, 95% CI −1.64 to 2.54) [60]. Based on the quality of the evidence and the safety profile of the medication, the expert panel conditionally recommended the use of MMF for the treatment of skin involvement.

Autologous stem cell transplantation (ASCT) compared to CYC was evaluated in three RCTs, which used different protocols [61–63]. The results of the meta-analysis showed a significant mRSS reduction in favor of ASCT (MD −12.0, 95% CI −15.24 to −8.64). Considering this moderate quality of evidence, the expert panel strongly recommended ASCT for the treatment of skin involvement in patients with progressive skin thickening refractory to previous immunosuppressive treatment and in patients with early dcSSc with several risk factors for worse prognosis.

Three RCTs evaluated rituximab (RTX) compared to placebo in the treatment of cutaneous involvement and demonstrated a 6.06-point reduction in mRSS (95% CI −10.51 to −1.61) [17, 64, 65]. Another RCT compared RTX (2 doses of 1000 mg at days 0 and 15) to intravenous CYC (monthly pulses of 500 mg/m² for 6 months) in 60 patients with dcSSc, with a decrease of 6.23 mRSS points in favor of RTX (95% CI −10.78 to −1.68) [66]. In the face of very low to low quality of evidence and considering the costs and availability of RTX, the panelists established a weak recommendation in favor of RTX compared to placebo and CYC for treating SSc cutaneous involvement.

The effect of tocilizumab (TCZ) on cutaneous involvement was evaluated as a primary outcome in two RCTs (faSScinate and focuSSed trials) including 294 patients with early and progressive dcSSc and altered inflammatory markers [67, 68]. There was a decrease of only 2.21 points in the mRSS (95% CI −4.08 to −0.34), with a quality of evidence rated as moderate. Considering the small magnitude of the effect, costs and access to medication, the expert panel strongly recommended against the use of TCZ for the treatment of skin fibrosis.

Another four RCTs evaluated the use of abatacept [69, 70], nintedanib (NTD) [71] and pirfenidone (PFD) [72] for the treatment of cutaneous involvement compared with placebo, and these drugs were strongly recommended against by panelists due to a lack of clinical efficacy based on moderate to high quality of evidence. Details on these studies are shown in Supplementary Table 7.

#### Recommendation 4

MTX, MMF and CYC are recommended as first-line therapies for the treatment of skin fibrosis. RTX may be an option in selected cases. ASCT is strongly recommended for the treatment of progressive and refractory cases.

### What is the evidence for the treatment of interstitial lung disease associated with SSc (SSc-ILD)?

SSc-ILD is present in 50–65% of patients and represents the leading cause of death in SSc [2, 8, 73]. While some patients will only have subclinical disease, many patients will have disease progression at different rates, especially in the first five years of the disease in the most severe cases [74], with such progression frequently associated with positive anti-Scl70 and African descent [75, 76]. Due to its prognostic importance, the lung is the only organ whose main manifestations were included in the 2013 SSc classification criteria [77].

In addition to the pharmacological treatment and in order to mitigate factors that could aggravate SSc-ILD, some interventions might be helpful, such as: updated vaccination (specially against pulmonary pathogens—pneumococci, influenza and coronavirus) and proper management of esophageal reflux to prevent aspiration [78]. Also, pulmonary rehabilitation and oxygen supplementation if hypoxia is present can lead to respiratory symptoms improvement [78, 79].

One RCT (SLS I) evaluated CYC at an oral dose ≤2 mg/kg/day compared to placebo for the treatment of SSc-ILD. The MD of the forced vital capacity (FVC) was 2.0% in favor of CYC (95% CI −1 to 4%) [57]. Another study, randomized but not blinded, compared the use of CYC (2 mg/kg/day for 12 months, with subsequent maintenance at 1 mg/kg/day) with AZA (2.5 mg/kg/day for 12 months with subsequent maintenance at 2 mg/kg/day) in 60 SSc patients. At the end of treatment, there was no variation in either the FVC or the diffusion capacity of carbon monoxide (DLCO) in the CYC group, but there was a statistically significant decrease in the FVC in the AZA group at as early as six months of treatment [58]. Considering the quality of the evidence found and the safety profile of the medication, the panel conditionally recommended the use of CYC in the treatment of SSc-ILD.

The SLS II evaluated MMF (target dose of 1.5 g twice daily for 24 months) compared with oral CYC (dose of 2 mg/kg/day for 12 months, followed by placebo for 12 months). There was a significant improvement in the post hoc analysis in the primary outcome (FVC) in both CYC (2.88%, 95% CI 1.19–4.58%) and MMF (2.19%, 95% CI 0.53–3.84%), with an MD of CVF between groups of 1% (95% CI −3 to 2%). However, the group using MMF had better tolerability and lower treatment failure [60]. Another study evaluating the efficacy of MMF at a dose of 2 g/day compared to placebo for six months in 41 patients with FVC ≥ 70% showed an MD in FVC of −6% (95% CI −10 to −2%) [59]. Therefore, the expert panel conditionally recommended the use of MMF in the treatment of SSc-ILD.

Recent studies have evaluated the efficacy of biologic agents, such as RTX and TCZ, in the treatment of SSc-ILD. A small randomized proof-of-principle study compared eight patients using RTX (two cycles, at baseline and after 24 weeks, each cycle consisting of four weekly doses of 375 mg/m^2^) associated with standard treatment with a control group of six patients who received only standard treatment; significant improvements were observed in the RTX group with respect to FVC (10.25% compared to a 5.4% deterioration in controls, *p* = 0.002) and DLCO (19.46% compared to a 7.5% deterioration in the control group, *p* = 0.023) [65]. Another small, double-blind RCT evaluated 16 patients with early SSc (< two years of disease), with the RTX group receiving 1000 mg infusions on days one and 15 as induction treatment and a single infusion of 1000 mg after six months, while the other group received placebo infusions; analyses of the extent of ILD on high-resolution chest tomography (HRCT) and on FVC showed slightly better results in the RTX group, but they were not statistically significant [64]. A RCT of 60 patients with dcSSc compared monthly pulses of CYC (500 mg/m^2^) with RTX (1000 mg in two doses, on days 1 and 15); a significant improvement in %FVC was observed in the RTX group (6.22%) when compared to the CYC group (−1.19; *p* = 0.03) [66]. Recently, the DESIRES study compared the use of RTX (at a dose of 375 mg/m^2^ IV, once per week for 4 consecutive weeks) with placebo for 24 weeks, in 56 Japanese patients with SSc. In the group using RTX, the mean %FVC improvement was 0.02% at week 24, while patients using placebo in the double-blind phase decreased their mean %FVC by −2.60% [17]. In light of the consistency of data from the DESIRES study, RTX has been approved for use in the treatment of SSc in Japan, and it was conditionally recommended by the Brazilian expert panel as an option for the treatment of SSc-ILD in patients who do not respond to the use of immunosuppressants.

TCZ was evaluated in two RCTs including 271 patients in total, which enrolled only patients with early, progressive, and diffuse skin disease. In both studies, subcutaneous TCZ at a dose of 162 mg weekly was compared against placebo for 48 weeks [67, 68]. The MD of %FVC at 48 weeks was 4% (95% CI 2–6%) in favor of the TCZ group [67, 68]. Although lung assessment was only a secondary outcome in the RCT analysis, TCZ has been approved for the treatment of SSc by the Food and Drug Administration (FDA) in the United States of America (USA), and it was conditionally recommended by the Brazilian panel as an option for the treatment of SSc-ILD in patients who do not respond to the use of immunosuppressants.

A new group of drugs with an important role in SSc-ILD are antifibrotics, such as NTD and PFD. An RCT compared the use of NTD, at a dose of 150 mg twice daily, with placebo in a group of 576 patients with SSc-ILD; 51.9% of these patients had dcSSc, and 48.4% were also receiving MMF at baseline. The primary endpoint was the adjusted annual rate of change in FVC after 48 weeks of treatment, which showed a lower decline in the NTD group (−52.4 ml) than in the placebo group (−93.3 ml) (95% CI 2.9–79 ml; *p* = 0.04) [71]. Based on these data, NTD has been approved for the treatment of SSc by the FDA in the USA and by the European Agency for the Evaluation of Medical Products (EMEA) in Europe. Due to the low quality of evidence and cost of the drug, it was conditionally recommended by the Brazilian expert panel as an option for the treatment of SSc-ILD.

The three RCTs comparing ASCT to CYC also evaluated the effect of stem cell transplantation on lung function, demonstrating an improvement in %FVC in favor of ASCT (MD 10%, 95% CI 4–16%) [61–63]. Thus, given the high cost, safety profile (increased treatment-related mortality in the first year after treatment) and side effects (severe or life-threatening adverse events), ASCT was conditionally recommended for selected and refractory cases of SSc-ILD, and should be early considered for patients at higher risk of progressive SSc-ILD.

Further details about the studies evaluating therapies for SSc-ILD can be found in Supplementary Table 8.

#### Recommendation 5

The expert panel conditionally recommends the use of CYC and MMF as first-line treatment for SSc-ILD. NTD, RTX and TCZ should be considered second-line options for the pharmacological treatment of SSc-ILD. ASCT should be considered for selected cases that are refractory to other treatment options.

### What therapeutic evidence is available for the treatment of gastrointestinal involvement (gastroesophageal reflux disease—GERD, gastrointestinal dysmotility, and small intestine bacterial overgrowth—SIBO) related to SSc?

Gastrointestinal (GI) involvement is ubiquitous in SSc patients, affecting up to 90% of patients [80]. Any part of the GI tract can be involved, and this is a very heterogeneous manifestation. Symptoms include GERD, dysphagia, diarrhea, SIBO, constipation and fecal incontinence [81]. Patients with severe involvement of the gastrointestinal tract have a higher risk of death [82]. Currently, there are no approved therapies directed for SSc-GI involvement, and there is no evidence that immunosuppression is effective in treating GI complications.

Prucalopride, a prokinetic with increased selectivity for the serotonin (5-HT_4_) receptor, was evaluated in one RCT [83]. The use of prucalopride 2 mg/day against placebo for one month in 29 SSc patients improved GI symptoms measured by the University of California Los Angeles Scleroderma Clinical Trials Consortium Gastrointestinal Tract Instrument 2.0 (UCLA GIT 2.0) questionnaire (SMD −0.17, 95% CI −0.2 to 0.1). Prucalopride has also been shown to increase bowel motility and to improve symptoms of constipation. Consequently, the expert panel conditionally recommended the use of prucalopride in SSc patients with SSc-GI involvement, especially for constipation.

The use of probiotics was evaluated in two RCTs [84, 85]. Both evaluated the efficacy of probiotics in improving GI symptoms measured by the UCLA GIT 2.0 questionnaire compared to placebo in SSc patients (for 60 days and eight weeks, respectively), but no significant improvement was observed (SMD −0.02 points, 95% CI −0.1 to 0.1). Hence, the expert panel conditionally recommended against the use of probiotics in SSc-GI. Specific details on the outcomes, evaluation instruments, measures of effect, certainty of the evidence, recommendation and agreement of these studies are described in Supplementary Table 9.

Although RCTs evaluating new therapies for SSc-GI are ongoing, we still have no pharmacological treatment specifically approved for SSc-GI, and therefore, we reinforce the SBR recommendations for the management of SSc made in 2013 [12]. In that manner, in addition to lifestyle modifications, such as avoiding triggering foods, eating small portions and elevating the head of the bed [78, 80–82], the use of proton pump inhibitors (PPIs) improves reflux esophagitis and GERD symptoms, and prokinetic agents, such as metoclopramide, domperidone, and octreotide, should be used to improve symptoms related to GI dysmotility. For SIBO, rotating antibiotics are recommended based on expert opinion. Nutritional support can be useful for patients with severe malnutrition [12]. These recommendations are in accordance with other guidelines for the management of SSc-GI [3].

#### Recommendation 6

For the pharmacological treatment of lower GI involvement/ constipation, the expert panel conditionally recommends the use of prucalopride. PPIs for GERD symptoms, other prokinetics for GI dysmotility and rotating antibiotics for SIBO are also recommended, based on expert opinion.

### What treatments are beneficial in the management of scleroderma renal crisis (SRC)?

SRC is the most important renal complication in SSc, affecting 5–10% of patients, especially those at higher risk, including patients with early dcSSc, rapidly progressive skin disease, presence of anti-RNA polymerase III and glucocorticoid therapy [86, 87]. SRC is characterized by acute hypertension and kidney injury, is considered a medical emergency and requires close monitoring and early identification [86, 87]. Regular blood pressure monitoring plays a critical role, especially in those patients with a higher risk of SRC. Prompt and aggressive blood pressure control with angiotensin-converting enzyme (ACE) inhibitors is mandatory, as the beneficial use of ACE inhibitors for SRC has already been demonstrated in cohort studies [3, 88, 89]. The experts recommend adding other antihypertensive agents (CCB, angiotensin receptor blockers, alpha and beta blockers) as required for blood pressure control if the patient’s blood pressure is not restored over 48–72 h. Renal substitutive therapy might be indicated for some patients. Due to the lack of RCTs on SRC, we reinforced the 2013 SBR recommendation for SRC treatment [12].

#### Recommendation 7

SSc patients presenting with SRC should be treated immediately with ACE inhibitors to control blood pressure and minimize kidney injury. Renal substitutive therapy and kidney transplantation might be indicated for those who do not recover renal function.

### What treatments are beneficial in the management of musculoskeletal manifestations (arthritis and/or myositis) in patients with SSc?

Musculoskeletal manifestations, including polyarthralgia, chronic arthritis, joint contractures, tendon friction rubs, and myositis, are common in patients with SSc and might impact the patient’s quality of life [7, 90]. As no placebo-controlled RCTs evaluating the treatment of these manifestations in SSc were found, recommendations were based on the opinion of the expert panel and data from cohort studies.

For the treatment of inflammatory arthritis, the therapeutic arsenal includes low-dose corticosteroids (defined as ≤ 15 mg/day of prednisone or equivalent [91]), MTX, and hydroxychloroquine. Nontumor necrosis factor biologics, especially RTX and TCZ, could be a promising option for refractory cases, but more data are necessary, especially from controlled studies in this population [90, 92]. Tofacitinib was also evaluated in one open-label clinical trial, and further controlled studies are needed [93].

For the pharmacological treatment of myositis, low-dose corticosteroids and MTX should be considered as treatment options, and RTX and IV immunoglobulin might be useful for refractory cases [92, 94]. Precaution with corticosteroid use is mandatory due to the increased risk of SRC [95].

#### Recommendation 8

Based on the expert panel opinion, the pharmacological treatment of inflammatory arthritis includes low-dose corticosteroids, MTX, and hydroxychloroquine. For the pharmacological treatment of myositis, low-dose corticosteroids and MTX should be considered a treatment option; RTX and IV immunoglobulin might be useful for severe and refractory cases.

## Conclusion

During the last decade, we have evolved in the comprehension of the pathogenic mechanisms of SSc, and therefore, many studies evaluating new therapeutic options have been conducted or are ongoing. Significant advances have been made and have led to a better prognosis for patients with SSc. The recommendations presented here include current scientific evidence revised under GRADE methodology and the experience of an expert panel aimed at guiding the management of SSc patients. The benefits of nonpharmacological approaches, such as physiotherapy, exercises and patient education, should be highlighted, but they were beyond the scope of this project. Guidelines for the treatment of SSc should be periodically updated as new scientific data from future studies continue to emerge.

## Electronic supplementary material

Below is the link to the electronic supplementary material.

Supplementary Material 1

## Data Availability

All data generated or analysed during this study are included in this published article and its supplementary information files.

## Abbreviations

ACE: Angiotensin-converting enzyme
ASCT: Autologous stem cell transplantation
AZA: Azathioprine
CCB: Calcium channel blocker
CI: Confidence interval
CYC: Cyclophosphamide
dcSSc: Diffuse cutaneous systemic sclerosis
DLCO: Diffusion capacity of the lungs for carbon monoxide
DU: Digital ulcer
EMEA: European Agency for the Evaluation of Medical Products
FDA: Food and Drug Administration
FVC: Forced vital capacity
GERD: Gastroesophageal reflux disease
GRADE: Grading of Recommendations Assessment, Development and Evaluation
HRCT: High-resolution chest tomography
ILD: Interstitial lung disease
ITT: Intention-to-treat
IV: Intravenous
lcSSc: Limited cutaneous systemic sclerosis
MD: Mean difference
MeSH: Medical Subject Heading
MMF: Mycophenolate mofetil
mRSS: Modified Rodnan skin score
MTX: Methotrexate
NTD: Nintedanib
PAH: Pulmonary arterial hypertension
PDE-5: Phosphodiesterase type 5
PFD: Pirfenidone
PICO: Patient/Population, Intervention, Comparison, Outcome
PPI: Proton pump inhibitors
PRISMA: Preferred Reporting Items for Systematic Reviews and Meta-Analyses
RCS: Raynaud’s Condition Score
RCT: Randomized clinical trial
RP: Raynaud’s phenomenon
RR: Risk ratio
RTX: Rituximab
SBR: Brazilian Society of Rheumatology
SD: Standard deviation
SIBO: Small intestine bacterial overgrowth
SE: Standard error
SLS: Scleroderma Lung Study
SMD: Standardized mean difference
SRC: Scleroderma renal crisis
SSc: Systemic sclerosis
TCZ: Tocilizumab
UCLA GIT: University of California Los Angeles Scleroderma Clinical Trials Consortium Gastrointestinal Tract Instrument
VAS: Visual Analogue Scale
