# Supplementary material for: 2023 Brazilian Society of Rheumatology guidelines for the treatment of systemic sclerosis
Source: Adv Rheumatol. 2024 Jul 10;64:52. doi: 10.1186/s42358-024-00392-w (PMC13488852; doi:10.1186/s42358-024-00392-w)
Supplement: Supplementary file 1 [file 42358_2024_392_MOESM1_ESM.docx]

**2023 Brazilian Society of Rheumatology guidelines for the treatment of systemic sclerosis**

-- **Supplementary material** –

Eleven clinical questions regarding SSc treatment were drafted by a core leadership team, consisting of four rheumatologists with expertise in SSc (C.K., S.O.M.D., A.T.D., and P.D.S.B.) according to the Patient/Population, Intervention, Comparison, Outcome (PICO) strategy (Supplementary chart 1). The clinical questions were revised and validated in a virtual meeting by a panel of experts, consisting of the 23 rheumatologists of the SBR SSc Committee. The PICO question regarding physical rehabilitation for musculoskeletal involvement in SSc patients was discarded by the expert panel because this work aimed to review pharmacological interventions. The PICO question about PAH was later discarded based on the presence of specific guidelines recently published [1]. The PICO question about cardiac involvement was discarded by the expert panel due to the lack of scientific evidence for tis management. As no placebo-controlled clinical trials for the treatment of scleroderma renal crisis (SRC) and musculoskeletal involvement were found, and due to the importance of these two clinical questions, the authors elaborated these two recommendations based on case controlled or head-to-head studies and on the expert opinion of the voting panel. The core leadership team also prespecified the outcomes that were considered critical or important for each PICO question for the systematic literature review. The methodological details, including PICO questions, outcomes and the search strategy for the systematic review are available in the Supplementary material (Supplementary tables 1 and 2).

**Supplementary chart 1.** Clinical questions.

1. What is the evidence for the pharmacological treatment of Raynaud’s phenomenon in patients with systemic sclerosis?

2. What evidence is available for the treatment of digital ulcers (DUs) in patients with SSc?

3. What evidence is available for pharmacological management in the prevention of DU recurrence in patients with SSc?

4. What is the evidence for the treatment of skin involvement in patients with systemic sclerosis?

5. What is the evidence for the treatment of interstitial lung disease associated with systemic sclerosis?

6. What therapeutic evidence is available for the treatment of gastrointestinal involvement (gastroesophageal reflux disease, gastrointestinal dysmotility, and small intestine bacterial overgrowth) related to systemic sclerosis?

7. What treatments are beneficial in the management of scleroderma renal crisis?

8. What treatments are beneficial in the management of musculoskeletal manifestations (arthritis and/or myositis) in patients with systemic sclerosis?

**Supplementary table 1.** PICO strategy for identification of keywords for the systematic literature review.

| **Patient/population** | **Intervention** | **Comparison** | **Outcome** |
| --- | --- | --- | --- |
| Systemic sclerosis, scleroderma, Raynaud’s phenomenon | Calcium channel blocker (nifedipine, diltiazem, amlodipine)  Phosphodiesterase-5 inhibitors (sildenafil, tadalafil)  Prostanoids (iloprost, alprostadil)  Endothelin receptor antagonists (bosentan)  Fluoxetine  Atorvastatin  Angiotensin converting enzyme inhibitors (captopril, enalapril, quinapril)  Angiotensin II-receptor antagonists (losartan) | Placebo | Improvement (frequency/severity/duration of Raynaud's phenomenon)  Side effects |
| Systemic sclerosis, scleroderma, digital ulcer | Phosphodiesterase-5 inhibitors (sildenafil, tadalafil)  Prostanoids (iloprost, alprostadil)  Endothelin receptor antagonists (bosentan, macitentan)  Atorvastatin  Antiplatelet drugs | Placebo | Number of ulcers  Healing  Healing time  Side effects |
| Systemic sclerosis, scleroderma, digital ulcer | Phosphodiesterase-5 inhibitors (sildenafil, tadalafil)  Prostanoids (iloprost, alprostadil)  Endothelin receptor antagonists (bosentan, macitentan)  Atorvastatin  Antiplatelet drugs | Placebo | Recurrence of new ulcers  Presence of new ulcers  Side effects |
| Systemic sclerosis, scleroderma, skin involvement/ thickness/ fibrosis | Cyclophosphamide  Mycophenolate mofetil  Methotrexate  Abatacept  Riociguat  Tocilizumab  Rituximab  Nintedanib  Lenabasum  Haematopoietic stem cell transplantation  Imatinib | Placebo  Cyclophosphamide  Azathioprine  Methotrexate | Improvement  Modified Rodnan skin score  Side effects |
| Systemic sclerosis, scleroderma, interstitial lung disease | Cyclophosphamide  Mycophenolate mofetil  Azathioprine  Tocilizumab  Rituximab  Nintedanib  Pirfenidone  Steroids  Haematopoietic stem cell transplantation  Lung transplantation | Placebo  Cyclophosphamide  Azathioprine | Lung function test  Forced vital capacity  Mortality  Side effects |
| Systemic sclerosis, scleroderma, gastroesophageal reflux, hypomotility and/or dysmotility, small intestine bacterial overgrowth | Proton pump inhibitors  Prokinetic drugs (domperidone, metoclopramide, cisapride, erythromicin)  Alginic acid  Baclofen  Buspirone  Intravenous immunoglobulin  Intermittent or rotating antibiotics  Surgery | Placebo | Improvement of gastrointestinal symptoms  Prevention of esophageal ulcers and strictures  UCLA GIT questionnaire  Side effects |

**Supplementary table 2**. Outcomes considered for each SSc manifestation evaluated.

| Clinical manifestation of SSc | Outcome |
| --- | --- |
| Raynaud’s phenomenon | Frequency, pain and intensity of RP attack  Visual Analogue Scale  Raynaud’s Condition Score |
| Digital ulcers | Healing  Healing time  Number of digital ulcers |
| Digital ulcers (recurrence) | Recurrence/presence of new ulcers |
| Skin thickness | Modified Rodnan skin score |
| Interstitial lung disease | Lung function test  Forced vital capacity  Mortality |
| Gastrointestinal involvement | Improvement of symptoms  UCLA GIT questionnaire  Prevention of peptic ulcers and stenosis |

RP: Raynaud’s phenomenon; SSc: systemic sclerosis; UCLA GIT: University of California Los Angeles Scleroderma Clinical Trial Consortium Gastrointestinal Tract Instrument.

**Supplementary data – Materials and methods**

## 1. *Eligibility criteria*

### a. *Studies*

All eligible randomized clinical controlled trials (RCTs), published in full or as an abstract were included. There was no restriction regarding the language of the publication.

### b. *Participants*

Participants should be adults diagnosed with SSc.

### c. *Types of interventions*

Studies comparing any active pharmacological intervention, used alone or in association, in any dose, to other active pharmacological intervention, used alone or in association or placebo were eligible to be included in the systematic revision. Interventions like stem-cell transplantation, adipose tissue-derived transplantation, botulinic toxin injection, and probiotics were also eligible.

2. *Data extraction*

Two reviewers (V.T.C. and A.R.) extracted data from methods (study design and definition), identification (source of sponsorship, country and author details such as name, institution, e-mail and address), participant characteristics (number of randomized participants, number of participants analyzed, and number of patients lost to follow-up and reasons described; characteristics of baseline data; inclusion and exclusion criteria), interventions (number of participants within each intervention group, description of intervention, dose, frequency, route of administration and length of treatment period), outcome measures (type of outcome, report, scale amplitude, unit of measure, direction, and observations), and any other relevant information using an extraction form in Excel software (Supplementary table 3). Disagreements between the two reviewers were discussed until a consensus was reached or resolved by a third reviewer (A.C.P.). Data was exported by one reviewer (V.T.C.) to the Review Manager 5 software to prevent inconsistencies.

**Supplementary table 3**. Search strategy used in each database.

| **Database** | **Search strategy** |
| --- | --- |
| **CENTRAL** | #1. MeSH descriptor: [Scleroderma, Systemic] explode all trees  #2. (Systemic Sclerosis) OR (Sclerosis, Systemic) OR (Systemic Scleroderma)  #3. #1 AND #2  Filter: Trials |
| **MEDLINE (PubMed)** | #1. " Scleroderma, Systemic "[Mesh] OR (Systemic Sclerosis) OR (Sclerosis, Systemic) OR (Systemic Scleroderma)  #2. ((clinical[Title/Abstract] AND trial[Title/Abstract]) OR clinical trials as topic[MeSH Terms] OR clinical trial[Publication Type] OR random*[Title/Abstract] OR random allocation[MeSH Terms] OR  therapeutic use[MeSH Subheading])  #3. #1 AND #2 |
| **Embase (Elsevier)** | #1. 'systemic sclerosis'/exp OR 'generalised scleroderma' OR 'generalized scleroderma' OR 'progressive scleroderma' OR 'progressive sclerodermia' OR 'progressive sclerosis, systemic' OR 'progressive systemic sclerosis' OR 'scleroderma, generalised' OR 'scleroderma, generalized' OR 'scleroderma, progressive' OR 'scleroderma, systemic' OR 'sclerosis, progressive systemic' OR 'sclerosis, systemic' OR 'sclerosis, systemic progressive' OR 'systemic progressive sclerosis' OR 'systemic scleroderma' OR 'systemic sclerosis, progressive'  #2. 'crossover procedure':de OR 'double-blind procedure':de OR 'randomized controlled trial':de OR 'single-blind procedure':de OR (random* OR factorial* OR crossover* OR cross NEXT/1 over* OR placebo* OR doubl* NEAR/1 blind* OR singl* NEAR/1 blind* OR assign* OR allocat* OR volunteer*):de,ab,ti  #3. #1 AND #2  #4. #3 AND [embase]/lim NOT ([embase]/lim AND [medline]/lim) |
| **Portal Regional BVS** | #1. MH:"Escleroderma Sistêmico" OR (Escleroderma Sistêmico) OR (Scleroderma, Systemic) OR (Esclerodermia Sistémica) OR (Esclerose Sistêmica) OR MH:C17.300.799$ OR MH:C17.800.784$  Filter: Lilacs |

3. *Risk of bias assessment*

The two reviewers (V.T.C. and A.R.) independently assessed the risk of bias of each included study using the Cochrane 'Risk of Bias' tool (RoB2) [2]. All disagreements were resolved by consensus, using the following definitions in assessing the risk of bias: bias arising from the randomization process; bias due to deviations from planned interventions; bias due to missing outcome data; bias in measuring the outcome; bias in the selection of reported results. The evaluation focused on the interference effects to interventions at baseline (the intention-to-treat effect). For the signaling questions within each domain for each outcome, one of the five possible answers was provided in the RoB2 tool ('Yes', 'Probably yes', 'No', 'Probably no' and 'No information'), judging as 'Low Risk of Bias', 'Some Concerns' or 'High Risk of Bias' according to the result of the algorithm.

4. *Measures of treatment effect and heterogeneity*

Data were summarized using meta-analyses by generic inverse variance or inverse variance method and random effects model. As some included trials presented the outcome measures in different units of measurement, the standardized mean difference (SMD) with the respective SE for each included trial was used, and then the pooled data were expressed as a difference of SMD and 95% CI. In supplementary tables 4-9, the SMD was further converted to typical scales as previously recommended [3].

When trials reported median and interquartile range or range, the mean and SD were estimated according to Wan's method. For dichotomous outcomes, the number of events and the number of participants in each intervention group of the included trials were extracted. The risk ratio (RR) was summarized with the respective 95% CI in random effects meta-analyses. No additional subgroup analyses were performed.

The unit of analysis considered was each participant (participants were randomized individually). Some included studies reported more than two driving arms, and in this case, data were extracted pairwise. When multiple time points were reported in the same included study, data related to the longest follow-up were extracted.

A possible clinical heterogeneity was assessed considering participants, interventions, outcomes and study characteristics for the included trials. Statistical heterogeneity was visually inspected using forest plots and the Chi^2^ test was used considering a threshold value of p < 0.1 as an indicator of the presence of heterogeneity. In addition, the I^2^ statistic was used to describe the proportion of variation in effect estimates that is due to between-study variability rather than sampling error.

I^2^ statistic was performed according to previous recommendations [4]:

- 0 to 40%: may not be important;
- 30% to 60%: may represent moderate heterogeneity;
- 50% to 74%: may represent substantial heterogeneity;
- 75% to 100%: considerable heterogeneity.

#### 5. Summaries of evidence and quality of the evidence

Meta-analyses were performed if more than one trial was included in each comparison. The meta-analyses were performed by pooling the included studies only if participants, interventions and outcomes were similar enough to be pooled.

Tables of key findings were created for all outcomes based on all available comparisons. For the assessment of the certainty of evidence, two authors (V.T.C. and A.R.) used the approach proposed by the *GRADE Working Group* [5]. The *software* GRADEpro GDT was used to analyze the overall certainty of the evidence for each outcome and any disagreements were resolved by consensus. Evidence certainty was downgraded by one level for severe limitations or two levels for very severe limitations based on defined criteria (study limitations, inconsistency, indirect evidence, imprecision of estimates, and presence of publication bias). At the end, the quality of evidence was categorized into four categories: high, moderate, low, or very low (Supplementary chart 2). Each decision to downgrade the certainty of the evidence was justified in footnotes.

**Supplementary chart 2**. GRADE Working Group grades of certainty.

| **Certainty** |  |
| --- | --- |
| High | The authors have a lot of confidence that the true effect lies close to the estimated effect |
| Moderate | The authors are moderately confident that the true effect is probably close to the estimated effect |
| Low | The confidence in the estimated effect is limited: the true effect may be substantially different from the estimated effect |
| Very low | The authors have very little confidence in the estimated effect: the true effect is likely to be substantially different from the estimated effect |

**Supplementary data – Results**

**Supplementary table 4**. Interventions for the treatment of Raynaud’s phenomenon in systemic sclerosis.

| **Comparison (studies)** | **Outcome**  **(Number of participants/studies)** | **Instrument of evaluation** | **Measure of effect (95% CI)** | **Certainty of evidence** | **Recommendation** | **Agreement** |
| --- | --- | --- | --- | --- | --- | --- |
| PDE-5 inhibitors x Placebo | Frequency of RP attacks (N = 200/7 RCTs)^6-12^ | Mean of RP attacks per day | MD -0.74 attacks per day (- 1.37 to -0.12) | ⨁⨁⨁◯ Moderate ^a^ | **Strongly recommended** | 95.7% |
|  | Severity of RP attacks  (N = 348/8 RCTs)^6-13^ | Raynaud's Condition Score (0-10) | MD -0.48  (-1.79 to 0.77) | ⨁⨁◯◯ Low ^a,b^ |  |  |
|  | Duration of RP attacks  (N = 267/7 RCTs)^6-12^ | Minutes | MD -15.2 minutes (-23.4 to -6.6) | ⨁⨁⨁◯ Moderate ^a^ |  |  |
| Nifedipine x Placebo | Frequency of RP attacks (N = 50/4 RCTs)^14-17^ | Mean of RP attacks per week | MD -5.5 attacks (-11.5 to 0.4) | ⨁◯◯◯ Very low ^a,c,d^ | **Strongly recommended** | 87% |
|  | Severity of RP attacks  (N = 21/1 RCT)^15^ | Visual Analogue Scale (0-10 cm) | MD 2.4 cm  (-4.1 to -0.7) | ⨁◯◯◯ Very low ^c,e^ |  |  |
|  | Duration of RP attacks  (N = 10/1 RCT)^16^ | Minutes | MD -13.4 minutes (-40.4 to 13.7) | ⨁◯◯◯ Very low^c,e^ |  |  |
| Iloprost x Placebo | Frequency of RP attacks (N = 566/5 RCTs)^18-22^ | Mean of RP attacks per day | MD -0.9 attacks (-1.64 to -0.15) | ⨁◯◯◯ Very low ^c,f^ | **Conditionally recommended** | 91.3% |
|  | Severity of RP attacks  (N = 615/6 RCTs)^18-22^ | Raynaud's Condition Score (0-10) | MD -0.26 cm (-0.82 to 0.29) | ⨁◯◯◯ Very low ^a,c,g^ |  |  |
|  | Duration of RP attacks  (N = 496/5 RCTs)^18-20, 22, 23^ | Minutes | MD -26 minutes (-60 to 7) | ⨁◯◯◯ Very low ^a,h^ |  |  |
| Prazosin x Placebo | Frequency of RP attacks (N = 21/1 RCT)^15^ | Mean of RP attacks per week | MD -1.5 attacks (-6.6 to 3.6) | ⨁◯◯◯ Very low ^c,e^ | **Conditionally recommended against** | 85.7% |
|  | Severity of RP attacks  (N = 21/1 RCT)^15^ | Visual Analogue Scale (0-10 cm) | MD -0.8 cm (-2.3 to 0.7) | ⨁◯◯◯ Very low ^c,e^ |  |  |
| Atorvastatin x Placebo | Severity of RP attacks  (N = 24/1 RCT)^24^ | Raynaud's Condition Score (0-10) | MD -1.3  (-2.68 to 0.08) | ⨁◯◯◯ Very low ^c,e^ | **Strongly recommended against** | 91.3% |
| Bosentan x Placebo | Frequency of RP attacks (N = 17/1 RCT)^25^ | Mean percentage change in daily frequency of RP attacks | MD 27%  (-9 to 63) | ⨁◯◯◯ Very low ^c,e^ | **Strongly recommended against** | 91.3% |
|  | Severity of RP attacks  (N = 17/1 RCT)^25^ | Mean percentage change in Raynaud's Condition Score | MD 5%  (-39 to 49) | ⨁◯◯◯ Very low ^c,e^ |  |  |
|  | Duration of RP attacks  (N = 17/1 RCT)^25^ | Mean percentage change | MD 34%  (6 to 62) | ⨁◯◯◯ Very low ^c,e^ |  |  |
| Grafting of autologous adipose tissue x Placebo | Frequency of RP attacks (N = 39/1 RCT)^26^ | Mean of RP attacks per day | MD 0.8 attacks (-1.31 to 2.91) | ⨁⨁◯◯ Low^e^ | **Strongly recommended against** | 91.3% |
|  | Severity of RP attacks  (N = 39/1 RCT)^26^ | Raynaud's Condition Score (0-10) | MD 0.4 (-1.55 to 2.35) | ⨁⨁◯◯ Low^e^ |  |  |
|  | Duration of RP attacks  (N = 39/1 RCT)^26^ | Minutes | MD 7 minutes (-2.12 to 16.12) | ⨁⨁◯◯ Low^e^ |  |  |
| Selexipag x Placebo | Frequency of RP attacks  (N = 59/1 RCT)^27^ | Mean of RP attacks per week | MD +3.8 attacks (-2.6 to 10.2) | ⨁◯◯◯ Very low ^c,e^ | **Strongly recommended against** | 87% |
|  | Duration of RP attacks  (N = 38/1 RCT)^27^ | Minutes | MD -1.9 minutes (-16.1 to 12.3) | ⨁◯◯◯ Very low ^c,e^ |  |  |
| Botulinum toxin x Placebo | Severity of RP attacks  (N = 40/1 RCT)^28^ | Raynaud's Condition Score (0-10) | MD -0.39  (-1.61 to 0.83) | ⨁◯◯◯ Very low ^c,e^ | **Strongly recommended against** | 78.3% |
| * The risk in the intervention group (and its 95% confidence interval) is based on the assumed risk of the comparator group and the relative effect of the intervention (and its 95% CI).  a. Downgraded one level due to imprecision  b. Downgraded one level due to inconsistence (I^2^ = 44%)  c. Downgraded one level due to risk of bias  d. Downgraded two levels due to inconsistence (I^2^ = 82%)  e. Downgraded two levels due to imprecision  f. Downgraded two levels due to inconsistence (I^2^ = 79%)  g. Downgraded two levels due to inconsistence (I^2^ = 69%)  h. Downgraded two levels due to inconsistence (I^2^ = 76%)  CI: confidence interval; MD: mean difference; PDE-5: phosphodiesterase type 5; RCT: randomized controlled trials; RP: Raynaud’s phenomenon. | | | | | | |
|  | | | | | | |

**Supplementary table 5**. Interventions for the healing treatment of digital ulcers in systemic sclerosis.

| **Comparison (studies)** | **Outcomes (Number of participants/studies)** | **Instrument of evaluation** | **Measure of effect (95% CI)** | **Certainty of evidence** | **Recommendation** | **Agreement** |
| --- | --- | --- | --- | --- | --- | --- |
| Sildenafil x Placebo | Digital ulcers (healing)  (N = 83/1 RCT)^13^ | Mean number of ulcers per patient | MD 0.6 less ulcers per patient  (-1.56 to 0.36) | ⨁⨁◯◯ Low ^b^ | **Strongly recommended** | 100% |
| Grafting of autologous adipose tissue x Placebo | Digital ulcers (healing) (N = 77/2 RCTs)^26,29^ | Number of patients with healed ulcers | RR 2.91 (0.27 to 31.73) | ⨁◯◯◯ Very low ^b,c,d^ | **Conditionally recommended** | 95.7% |
| Iloprost x Placebo | Digital ulcers (healing) (N = 11/1 RCT)^15^ | Number of patients with healed ulcers | RR 8.12 (0.57 to 115.07) | ⨁◯◯◯ Very low ^e^ | **Conditionally recommended** | 95.7% |
| Selexipag x Placebo | Digital ulcers (healing) (N = 11/1 RCT)^27^ | Number of healed ulcers from baseline | RR 1.43 (0.76 to 2.71) | ⨁◯◯◯ Very low ^b,c^ | **Strongly recommended against** | 90.5% |
| Macitentan x Placebo | Digital ulcers (healing) (N = 533/2 RCTs)^30^ | Number of patients with healed ulcers | RR 0.86 (0.68 to 1.09) | ⨁⨁⨁◯ Moderate ^a^ | **Strongly recommended against** | 100% |
| Botulinum toxin x Placebo | Digital ulcers (healing) (N = 40/1 RCT)^28^ | Number of ulcers per group | MD 0.08 less ulcers  (-0.48 to 0.32) | ⨁◯◯◯ Very low ^b,c^ | **Strongly recommended against** | 85.7% |
| * The risk in the intervention group (and its 95% confidence interval) is based on the assumed risk of the comparator group and the relative effect of the intervention (and its 95% CI).  a. Downgraded one level due to imprecision  b. Downgraded two levels due to imprecision  c. Downgraded one level due to risk of bias  d. Downgraded two levels due to inconsistence (I^2^ = 84%)  e. Downgraded three levels due to imprecision  CI: confidence interval; MD: mean difference; RCT: randomized controlled trial; RR: risk ratio. | | | | | | |

**Supplementary table 6**. Interventions for the treatment of recurrent digital ulcers in systemic sclerosis.

| **Comparison (studies)** | **Outcome**  **(Number of participants/studies)** | **Instrument of evaluation** | **Measure of effect (95% CI)** | **Certainty of evidence** | **Recommendation** | **Agreement** |
| --- | --- | --- | --- | --- | --- | --- |
| Bosentan x Placebo | Digital ulcer (recurrence)  (N = 305/2 RCTs)^31,32^ | Mean of new ulcers per patient | MD 1.22 less new ulcers per patient (-1.58 to -0.87) | ⨁⨁⨁◯ Moderate ^a^ | **Strongly recommended** | 82.6% |
| Sildenafil x Placebo | Digital ulcer (recurrence)  (N = 75/1 RCT)^13^ | Number of patients with new ulcer | RR 0.55 (0.26 to 1.14) | ⨁⨁◯◯ Low ^b^ | **Strongly recommended** | 100% |
| Iloprost x Placebo | Digital ulcer (recurrence)  (N = 24/1 RCT)^20^ | Number of patients with new ulcer | RR 1.18 (0.30 to 4.72) | ⨁◯◯◯ Very low ^c^ | **Conditionally recommended** | 95.7% |
| Macitentan x Placebo | Digital ulcer (recurrence)  (N = 554/2 RCTs)^30^ | Number of patients with new ulcer | MD 0.19 new ulcers per patient (-0.13 to 0.51) | ⨁⨁⨁⨁ High | **Strongly recommended against** | 100% |
| Botulinum toxin x Placebo | Digital ulcer (recurrence)  (N = 18/1 RCT)^28^ | Risk of new ulcers development | RR 1.27 (0.68 to 2.37) | ⨁◯◯◯ Very low ^b,d^ | **Strongly recommended against** | 85.7% |
| * The risk in the intervention group (and its 95% confidence interval) is based on the assumed risk of the comparator group and the relative effect of the intervention (and its 95% CI).  a. Downgraded one level due to imprecision  b. Downgraded two levels due to imprecision  c. Downgraded three levels due to imprecision  d. Downgraded two levels due to risk of bias  CI: confidence interval; MD: mean difference; RCT: randomized controlled trial; RR: risk ratio. | | | | | | |
|  | | | | | | |

**Supplementary chart 7.** Interventions for the treatment of skin involvement in systemic sclerosis.

| **Comparison (studies)** | **Outcome**  **(Number of participants/studies)** | **Instrument of evaluation** | **Measure of effect (95% CI)** | **Certainty of evidence** | **Recommendation** | **Agreement** |
| --- | --- | --- | --- | --- | --- | --- |
| Methotrexate x Placebo | Skin fibrosis  (N = 100/2 RCTs)^33,34^ | Modified Rodnan Skin Score (0-78) | MD -5.17  (-10.08 to -0.13) | ⨁⨁⨁◯ Moderate ^a^ | **Strongly recommended** | 96% |
| Mycophenolate x Cyclophosphamide | Skin fibrosis (N = 106/1 RCT)^35^ | Modified Rodnan Skin Score (0-51) | MD 0.45  (-1.64 to 2.54) | ⨁⨁⨁◯ Moderate ^a^ | **Strongly recommended** | 78% |
| Stem cell transplantation x Cyclophosphamide | Skin fibrosis (N = 225/3 RCTs)^36-38^ | Modified Rodnan Skin Score (0-51) | MD -12 (-15.24 to -8.64) | ⨁⨁⨁◯ Moderate ^a^ | **Strongly recommended** | 78,3% |
| Cyclophosphamide x Placebo | Skin fibrosis (N = 145/1 RCT)^39^ | Modified Rodnan Skin Score (0-51) | MD -2.7  (-5.53 to 0.13) | ⨁⨁⨁◯ Moderate ^a^ | **Conditionally recommended** | 87% |
| Cyclophosphamide x Azathioprine | Skin fibrosis (N = 60/1 RCT)^40^ | Modified Rodnan Skin Score (0-51) | MD -9.67  (-9.98 to -9.36) | ⨁◯◯◯ Very low ^b,c^ | **Conditionally recommended** | 91% |
| Mycophenolate x Placebo | Skin fibrosis (N = 41/1 RCT)^41^ | Modified Rodnan Skin Score (0-51) | MD - 3,3 (-6.18 to -0.42) | ⨁⨁◯◯ Low ^c^ | **Conditionally recommended** | 83% |
| Rituximab x Placebo | Skin fibrosis (N = 84/3 RCTs)^42-44^ | Modified Rodnan Skin Score (0-51) | MD -6.06 (-10.51 to -1.61) | ⨁⨁◯◯  Low ^a,b^ | **Conditionally recommended** | 96% |
| Rituximab x Cyclophosphamide | Skin fibrosis (N = 60/1 RCT)^45^ | Modified Rodnan Skin Score (0-51) | MD -6.23  (-10.78 to -1.68) | ⨁◯◯◯ Very low ^b,c^ | **Conditionally recommended** | 100% |
| Abatacept x Placebo | Skin fibrosis (N = 98/2 RCTs)^46,47^ | Modified Rodnan Skin Score (0-51) | MD -1.89  (-5.02 to 1.24) | ⨁⨁⨁◯ Moderate ^a^ | **Strongly recommended against** | 96% |
| Nintedanib x Placebo | Skin fibrosis (N = 574/1 RCT)^48^ | Modified Rodnan Skin Score (0-51) | MD -0.21 (-0.94 to 0.52) | ⨁⨁⨁⨁ High | **Strongly recommended against** | 96% |
| Pirfenidone x Placebo | Skin fibrosis (N = 34/1 RCT)^49^ | Modified Rodnan Skin Score (0-51) | MD -1.5 (-4.64 to 1.64) | ⨁⨁◯◯ Low ^c^ | **Strongly recommended against** | 100% |
| Tocilizumab x Placebo | Skin fibrosis (N = 294/2 RCTs)^50,51^ | Modified Rodnan Skin Score (0-51) | MD 2.21  (-4.08 to -0.34) | ⨁⨁⨁◯ Moderate ^a^ | **Strongly recommended against** | 78% |
| * The risk in the intervention group (and its 95% confidence interval) is based on the assumed risk of the comparator group and the relative effect of the intervention (and its 95% CI).  a. Downgraded one level due to imprecision  b. Downgraded one level due to risk of bias  c. Downgraded two levels due to imprecision  CI: confidence interval; MD: mean difference; RCT: randomized controlled trial. | | | | | | |

**Supplementary table 8.** Interventions for the treatment of interstitial lung disease in systemic sclerosis.

| **Comparison (studies)** | **Outcome**  **(Number of participants/studies)** | **Instrument of evaluation** | **Measure of effect (95% CI)** | **Certainty of evidence** | **Recommendation** | **Agreement** |
| --- | --- | --- | --- | --- | --- | --- |
| Cyclophosphamide x Placebo | Forced vital capacity  (N = 145/1 RCT)^39^ | Percentage of predict | MD 2% higher (-1 to 4) | ⨁⨁◯◯ Low ^a,b^ | **Conditionally recommended** | 78% |
|  | Mortality  (N = 158/1 RCT)^39^ | Number of events | RR 1.00 (0.34 to 2.97) | ⨁◯◯◯ Very low ^a,c^ |  |  |
| Cyclophosphamide x Azathioprine | Forced vital capacity  (N = 60/1 RCT)^40^ | Percentage of predict | MD 14% higher  (14 to 15) | ⨁◯◯◯ Very low ^a,c^ | **Conditionally recommended** | 87% |
| Mycophenolate x Placebo | Forced vital capacity  (N = 41/1 RCT)^41^ | Percentage of predict | MD 6% lower (-10 to -2) | ⨁⨁◯◯ Low ^c^ | **Conditionally recommended** | 78.3% |
| Mycophenolate x Cyclophosphamide | Forced vital capacity  (N = 145/1 RCT)^35^ | Percentage of predict | MD 1% lower (-3 to 2) | ⨁⨁◯◯ Low ^c^ | **Conditionally recommended** | 91% |
|  | Mortality  (N = 158/1 RCT)^35^ | Number of events | RR 0.44 (0.16 to 1.17) | ⨁⨁◯◯ Low ^c^ |  |  |
| Nintedanib x Placebo | Forced vital capacity  (N = 614/2 RCTs)^48,52^ | Annual rate of decline in FVC | MD less 44.53 ml/year (-81.5 to -7.56) | ⨁⨁◯◯ Low ^a,b^ | **Conditionally recommended** | 87% |
|  | Mortality  (N = 746/2 RCTs)^48,52^ | Number of events | RR 0.93 (0.50 to 1.72) | ⨁⨁◯◯ Low ^c^ |  |  |
| Rituximab x Placebo | Forced vital capacity  (N = 84/3 RCTs)^42-44^ | Percentage of predict | MD 5% higher (1 to 8) | ⨁⨁◯◯ Low ^a,b^ | **Conditionally recommended** | 83% |
|  | Mortality  (N = 72/2 RCTs)^42-44^ | Number of events | No event in both groups | - |  |  |
| Rituximab x Cyclophosphamide | Forced vital capacity  (N = 60/1 RCT)^45^ | Percentage of predict | MD 7% higher (-1 to 16) | ⨁◯◯◯ Very low ^a,c^ | **Conditionally recommended** | 96% |
|  | Mortality  (N = 60/1 RCT)^45^ | Number of events | RR 1.00 (0.07 to 15.26) | ⨁◯◯◯ Very low ^a,c^ |  |  |
| Tocilizumab x Placebo | Forced vital capacity  (N = 271/2 RCTs)^50,51^ | Percentage of predict | MD 4% higher (2 to 6) | ⨁⨁⨁◯ Moderate ^b^ | **Conditionally recommended** | 91% |
|  | Mortality  (N = 297/2 RCTs)^50,51^ | Number of events | RR 1.03 (0.12 to 8.88) | ⨁⨁◯◯ Low ^c^ |  |  |
| Stem cell transplantation x Cyclophosphamide | Forced vital capacity  (N = 150/2 RCTs)^36-38^ | Percentage of predict | MD 10% higher (4 to 16) | ⨁⨁◯◯ Low ^a,b^ | **Conditionally recommended** | 96% |
|  | Mortality  (N = 250/3 RCTs)^36-38^ | Number of events | RR 0.74 (0.47 to 1.16) | ⨁⨁◯◯ Low ^a,b^ |  |  |
| Pirfenidone x Placebo | Forced vital capacity  (N = 34/1 RCT)^50^ | Percentage of predict | MD 6% higher (-1 to 14) | ⨁◯◯◯ Very low ^a,c^ | **Conditionally recommended against** | 78% |
|  | Mortality  (N = 34/1 RCT)^50^ | Number of events | No event in both groups | - |  |  |
| * The risk in the intervention group (and its 95% confidence interval) is based on the assumed risk of the comparator group and the relative effect of the intervention (and its 95% CI).  a. Downgraded one level due to risk of bias  b. Downgraded one level due to imprecision  c. Downgraded two levels due to imprecision  CI: confidence interval; FVC: forced vital capacity; MD: mean difference; RCT: randomized controlled trial; RR: risk ratio. | | | | | | |
|  | | | | | | |

**Supplementary table 9**. Interventions for the treatment of gastrointestinal involvement in systemic sclerosis.

| **Comparison (studies)** | **Outcome**  **(Number of participants/studies)** | **Instrument of evaluation** | **Measure of effect (95% CI)** | **Certainty of evidence** | **Recommendation** | **Agreement** |
| --- | --- | --- | --- | --- | --- | --- |
| Prucalopride x Placebo | Symptoms reduction  (N = 29/1 RCT)^53^ | UCLA GIT Questionnaire (0-3) | MD 0.17 points less (-0.2 to 0.1) | ⨁◯◯◯ Very low ^a,b^ | **Conditionally recommended** | 95.7% |
| Probiotics x Placebo | Symptoms reduction  (N = 113/2 RCTs)^54,55^ | UCLA GIT Questionnaire (0-3) | MD 0.02 points less (-0.1 to 0.1) | ⨁⨁◯◯ Low ^c,d^ | **Conditionally recommended against** | 91.3% |
| * The risk in the intervention group (and its 95% confidence interval) is based on the assumed risk of the comparator group and the relative effect of the intervention (and its 95% CI).  a. Downgraded one level due to risk of bias  b. Downgraded two levels due to imprecision  c. Downgraded one level due to inconsistence (I^2^ = 50%)  d. Downgraded one level due to imprecision  CI: confidence interval; MD: mean difference; RCT: randomized controlled trial; UCLA GIT: University of California Los Angeles Scleroderma Clinical Trial Consortium Gastrointestinal Tract Instrument. | | | | | | |
|  | | | | | | |

**References**

1. Humbert M, Kovacs G, Hoeper MM, Badagliacca R, Berger RMF, Brida M, et al; ESC/ERS Scientific Document Group. 2022 ESC/ERS Guidelines for the diagnosis and treatment of pulmonary hypertension. Eur Respir J. 2023;61(1):2200879. doi:10.1183/13993003.00879-2022.
2. Higgins JPT, Savović J, Page MJ, Elbers RG, Sterne JAC. Chapter 8: Assessing risk of bias in a randomized trial. *In*: Higgins JPT, Thomas J, Chandler J, Cumpston M, Li T, Page MJ, et al (editors). Cochrane Handbook for Systematic Reviews of Interventions version 6.3 (updated in February 2022). Cochrane, 2022. http://[www.training.cochrane.org/handbook](http://www.training.cochrane.org/handbook).
3. Schünemann HJ, Vist GE, Higgins JPT, Santesso N, Deeks JJ, Glasziou P, et al. Chapter 15: Interpreting results and drawing conclusions. *In*: Higgins JPT, Thomas J, Chandler J, Cumpston M, Li T, Page MJ, et al (editors). Cochrane Handbook for Systematic Reviews of Interventions version 6.3 (updated in February 2022). Cochrane, 2022. http://[www.training.cochrane.org/handbook](http://www.training.cochrane.org/handbook).
4. Deeks JJ, Higgins JPT, Altman DG (editors). Chapter 10: Analysing data and undertaking meta-analyses. *In*: Higgins JPT, Thomas J, Chandler J, Cumpston M, Li T, Page MJ, et al (editors). Cochrane Handbook for Systematic Reviews of Interventions version 6.3 (updated in February 2022). Cochrane, 2022. http://[www.training.cochrane.org/handbook](http://www.training.cochrane.org/handbook).
5. Schünemann H, Brożek J, Guyatt G, Oxman A. GRADE handbook for grading quality of evidence and strength of recommendations (updated in October 2013). The GRADE Working Group; 2013. http://[www.guidelinedevelopment.org/handbook](http://www.guidelinedevelopment.org/handbook).
6. Agarwal V, Ghosh P, Sharma A, Bhakuni DS, Kumar S, Singh UN, et al. Efficacy of Tadalafil in Raynaud's phenomenon secondary to systemic sclerosis: A double blind randomized placebo controlled parallel group multicentric study. Arthritis Rheum. 2010;62 Suppl 10:2086.[ABSTRACT]
7. Andrigueti FV, Ebbing PCC, Arismendi MI, Kayser C. Evaluation of the effect of sildenafil on the microvascular blood flow in patients with systemic sclerosis: a randomised, double-blind, placebo-controlled study. Clin Exp Rheumatol. 2017;35 Suppl 106(4):151-158.
8. Caglayan E, Axmann S, Hellmich M, Moinzadeh P, Rosenkranz S. Vardenafil for the treatment of Raynaud phenomenon: a randomized, double-blind, placebo-controlled crossover study. Arch Intern Med. 2012;172(15):1182-1184. doi:10.1001/archinternmed.2012.2271.
9. Fries R, Shariat K, von Wilmowsky H, Böhm M. Sildenafil in the treatment of Raynaud's phenomenon resistant to vasodilatory therapy. Circulation. 2005;112(19):2980-2985. doi:10.1161/CIRCULATIONAHA.104.523324.
10. Herrick AL, van den Hoogen F, Gabrielli A, Tamimi N, Reid C, O'Connell D, et al. Modified-release sildenafil reduces Raynaud's phenomenon attack frequency in limited cutaneous systemic sclerosis. Arthritis Rheum. 2011;63(3):775-782. doi:10.1002/art.30195.
11. Schiopu E, Hsu VM, Impens AJ, Rothman JA, McCloskey DA, Wilson JE, et al. Randomized placebo-controlled crossover trial of tadalafil in Raynaud's phenomenon secondary to systemic sclerosis. J Rheumatol. 2009;36(10):2264-2268. doi:10.3899/jrheum.090270.
12. Shenoy PD, Kumar S, Jha LK, Choudhary SK, Singh U, Misra R, et al. Efficacy of tadalafil in secondary Raynaud's phenomenon resistant to vasodilator therapy: a double-blind randomized cross-over trial. Rheumatology (Oxford). 2010;49(12):2420-2428. doi:10.1093/rheumatology/keq291.
13. Hachulla E, Hatron PY, Carpentier P, Agard C, Chatelus E, Jego P, et al; SEDUCE study group. Efficacy of sildenafil on ischaemic digital ulcer healing in systemic sclerosis: the placebo-controlled SEDUCE study. Ann Rheum Dis. 2016;75(6):1009-1015. doi:10.1136/annrheumdis-2014-207001.
14. Kahan A, Weber S, Amor B, Menkes CJ, Saporta L, Hodara M, et al. Calcium entry blocking agents in digital vasospasm (Raynaud's phenomenon). Eur Heart J. 1983;4 Suppl C:123-129. doi:10.1093/eurheartj/4.suppl_c.123.
15. Kahan A, Foult JM, Weber S, Amor B, Menkes CJ, Degeorges M. Nifedipine and alpha 1-adrenergic blockade in Raynaud's phenomenon. Eur Heart J. 1985;6(8):702-705. doi:10.1093/oxfordjournals.eurheartj.a061923.
16. Meyrick Thomas RH, Rademaker M, Grimes SM, MacKay A, Kovacs IB, Cook ED, et al. Nifedipine in the treatment of Raynaud's phenomenon in patients with systemic sclerosis. Br J Dermatol. 1987;117(2):237-241. doi:10.1111/j.1365-2133.1987.tb04122.x.
17. Rodeheffer RJ, Rommer JA, Wigley F, Smith CR. Controlled double-blind trial of nifedipine in the treatment of Raynaud's phenomenon. N Engl J Med. 1983;308(15):880-883. doi:10.1056/NEJM198304143081507.
18. Black CM, Halkier-Sørensen L, Belch JJ, Ullman S, Madhok R, Smit AJ, et al. Oral iloprost in Raynaud's phenomenon secondary to systemic sclerosis: a multicentre, placebo-controlled, dose-comparison study. Br J Rheumatol. 1998;37(9):952-960. doi:10.1093/rheumatology/37.9.952.
19. McHugh NJ, Csuka M, Watson H, Belcher G, Amadi A, Ring EF, et al. Infusion of iloprost, a prostacyclin analogue, for treatment of Raynaud's phenomenon in systemic sclerosis. Ann Rheum Dis. 1988;47(1):43-47. doi:10.1136/ard.47.1.43.
20. Wigley FM, Seibold JR, Wise RA, McCloskey DA, Dole WP. Intravenous iloprost treatment of Raynaud's phenomenon and ischemic ulcers secondary to systemic sclerosis. J Rheumatol. 1992;19(9):1407-1414.
21. Wigley FM, Wise RA, Seibold JR, McCloskey DA, Kujala G, Medsger TA Jr, et al. Intravenous iloprost infusion in patients with Raynaud phenomenon secondary to systemic sclerosis. A multicenter, placebo-controlled, double-blind study. Ann Intern Med. 1994;120(3):199-206. doi:10.7326/0003-4819-120-3-199402010-00004.
22. Wigley FM, Korn JH, Csuka ME, Medsger TA Jr, Rothfield NF, Ellman M, et al. Oral iloprost treatment in patients with Raynaud's phenomenon secondary to systemic sclerosis: a multicenter, placebo-controlled, double-blind study. Arthritis Rheum. 1998;41(4):670-677. doi:10.1002/1529-0131(199804)41:4<670::AID-ART14>3.0.CO;2-I.
23. Belch JJ, Capell HA, Cooke ED, Kirby JD, Lau CS, Madhok R, et al. Oral iloprost as a treatment for Raynaud's syndrome: a double-blind multicentre placebo-controlled study. Ann Rheum Dis. 1995;54(3):197-200. doi:10.1136/ard.54.3.197.
24. Domsic R, Laffoon M, Goundappa B, Medsger T, Lafyatis R, Wisniewski S. The effect of atorvastatin on microvascular endothelial function and Raynaud phenomenon in early diffuse scleroderma: Results of the “tamer” study. Arthritis Rheum. 2019;71 Suppl 10:1505-6. [ABSTRACT].
25. Nguyen VA, Eisendle K, Gruber I, Hugl B, Reider D, Reider N. Effect of the dual endothelin receptor antagonist bosentan on Raynaud's phenomenon secondary to systemic sclerosis: a double-blind prospective, randomized, placebo-controlled pilot study. Rheumatology (Oxford). 2010;49(3):583-587. doi:10.1093/rheumatology/kep413.
26. Daumas A, Magalon J, Jouve E, Casanova D, Philandrianos C, Abellan Lopez M, et al. Adipose tissue-derived stromal vascular fraction for treating hands of patients with systemic sclerosis: a multicentre randomized trial Autologous AD-SVF versus placebo in systemic sclerosis. Rheumatology (Oxford). 2022;61(5):1936-1947. doi:10.1093/rheumatology/keab584.
27. Denton CP, Hachulla É, Riemekasten G, Schwarting A, Frenoux JM, Frey A, et al; Raynaud Study Investigators. Efficacy and Safety of Selexipag in Adults with Raynaud's Phenomenon Secondary to Systemic Sclerosis: A Randomized, Placebo-Controlled, Phase II Study. Arthritis Rheumatol. 2017;69(12):2370-2379. doi:10.1002/art.40242.
28. Bello RJ, Cooney CM, Melamed E, Follmar K, Yenokyan G, Leatherman G, et al. The Therapeutic Efficacy of Botulinum Toxin in Treating Scleroderma-Associated Raynaud's Phenomenon: A Randomized, Double-Blind, Placebo-Controlled Clinical Trial. Arthritis Rheumatol. 2017;69(8):1661-1669. doi:10.1002/art.40123.
29. Del Papa N, Di Luca G, Andracco R, Zaccara E, Maglione W, Pignataro F, et al. Regional grafting of autologous adipose tissue is effective in inducing prompt healing of indolent digital ulcers in patients with systemic sclerosis: results of a monocentric randomized controlled study. Arthritis Res Ther. 2019;21(1):7. doi:10.1186/s13075-018-1792-8.
30. Khanna D, Denton CP, Merkel PA, Krieg T, Le Brun FO, Marr A, et al; DUAL-1 Investigators; DUAL-2 Investigators. Effect of Macitentan on the Development of New Ischemic Digital Ulcers in Patients with Systemic Sclerosis: DUAL-1 and DUAL-2 Randomized Clinical Trials. JAMA. 2016;315(18):1975-1988. doi:10.1001/jama.2016.5258.
31. Korn JH, Mayes M, Matucci-Cerinic M, Rainisio M, Pope J, Hachulla E, et al. Digital ulcers in systemic sclerosis: prevention by treatment with bosentan, an oral endothelin receptor antagonist. Arthritis Rheum. 2004;50(12):3985-3993. doi:10.1002/art.20676.
32. Matucci-Cerinic M, Denton CP, Furst DE, Mayes MD, Hsu VM, Carpentier P, et al. Bosentan treatment of digital ulcers related to systemic sclerosis: results from the RAPIDS-2 randomized, double-blind, placebo-controlled trial. Ann Rheum Dis. 2011;70(1):32-38. doi:10.1136/ard.2010.130658.
33. Pope JE, Bellamy N, Seibold JR, Baron M, Ellman M, Carette S, et al. A randomized, controlled trial of methotrexate versus placebo in early diffuse scleroderma. Arthritis Rheum. 2001;44(6):1351-1358. doi:10.1002/1529-0131(200106)44:6<1351::AID-ART227>3.0.CO;2-I.
34. van den Hoogen FH, Boerbooms AM, Swaak AJ, Rasker JJ, van Lier HJ, van de Putte LB. Comparison of methotrexate with placebo in the treatment of systemic sclerosis: a 24-week randomized double-blind trial, followed by a 24-week observational trial. Br J Rheumatol. 1996;35(4):364-372. doi:10.1093/rheumatology/35.4.364.
35. Tashkin DP, Roth MD, Clements PJ, Furst DE, Khanna D, Kleerup EC, et al; Sclerodema Lung Study II Investigators. Mycophenolate mofetil versus oral cyclophosphamide in scleroderma-related interstitial lung disease (SLS II): a randomized controlled, double-blind, parallel group trial. Lancet Respir Med. 2016;4(9):708-719. doi:10.1016/S2213-2600(16)30152-7.
36. Burt RK, Shah SJ, Dill K, Grant T, Gheorghiade M, Schroeder J, et al. Autologous non-myeloablative haemopoietic stem-cell transplantation compared with pulse cyclophosphamide once per month for systemic sclerosis (ASSIST): an open-label, randomized phase 2 trial. Lancet. 2011;378(9790):498-506. doi:10.1016/S0140-6736(11)60982-3.
37. Sullivan KM, Goldmuntz EA, Keyes-Elstein L, McSweeney PA, Pinckney A, Welch B, et al; SCOT Study Investigators. Myeloablative Autologous Stem-Cell Transplantation for Severe Scleroderma. N Engl J Med.;378(1):35-47. doi:10.1056/nejmoa1703327.
38. van Laar JM, Farge D, Sont JK, Naraghi K, Marjanovic Z, Larghero J, et al; EBMT/EULAR Scleroderma Study Group. Autologous hematopoietic stem cell transplantation vs intravenous pulse cyclophosphamide in diffuse cutaneous systemic sclerosis: a randomized clinical trial. JAMA. 2014;311(24):2490-2498. doi:10.1001/jama.2014.6368.
39. Tashkin DP, Elashoff R, Clements PJ, Goldin J, Roth MD, Furst DE, et al; Scleroderma Lung Study Research Group. Cyclophosphamide versus placebo in scleroderma lung disease. N Engl J Med. 2006;354(25):2655-2666. doi:10.1056/NEJMoa055120.
40. Nadashkevich O, Davis P, Fritzler M, Kovalenko W. A randomized unblinded trial of cyclophosphamide versus azathioprine in the treatment of systemic sclerosis. Clin Rheumatol. 2006;25(2):205-212. doi:10.1007/s10067-005-1157-y.
41. Naidu GSRSNK, Sharma SK, Adarsh MB, Dhir V, Sinha A, Dhooria S, Jain S. Effect of mycophenolate mofetil (MMF) on systemic sclerosis-related interstitial lung disease with mildly impaired lung function: a double-blind, placebo-controlled, randomized trial. Rheumatol Int. 2020;40(2):207-216. doi:10.1007/s00296-019-04481-8.
42. Boonstra M, Meijs J, Dorjée AL, Marsan NA, Schouffoer A, Ninaber MK, et al. Rituximab in early systemic sclerosis. RMD Open. 2017;3(2):e000384. doi:10.1136/rmdopen-2016-000384.
43. Daoussis D, Liossis SN, Tsamandas AC, Kalogeropoulou C, Kazantzi A, Sirinian C, et al. Experience with rituximab in scleroderma: results from a 1-year, proof-of-principle study. Rheumatology (Oxford). 2010;49(2):271-280. doi:10.1093/rheumatology/kep093.
44. Ebata S, Yoshizaki A, Oba K, Kashiwabara K, Ueda K, Uemura Y, et al. Safety and efficacy of rituximab in systemic sclerosis (DESIRES): a double-blind, investigator-initiated, randomized, placebo-controlled trial. Lancet Rheumatol 2021;3:e489-97. doi:10.1016/S2665-9913(22)00131-X.
45. Sircar G, Goswami RP, Sircar D, Ghosh A, Ghosh P. Intravenous cyclophosphamide vs rituximab for the treatment of early diffuse scleroderma lung disease: open label, randomized, controlled trial. Rheumatology (Oxford). 2018;57(12):2106-2113. doi:10.1093/rheumatology/key213.
46. Chakravarty EF, Martyanov V, Fiorentino D, Wood TA, Haddon DJ, Jarrell JA, et al. Gene expression changes reflect clinical response in a placebo-controlled randomized trial of abatacept in patients with diffuse cutaneous systemic sclerosis. Arthritis Res Ther. 2015;17(1):159. doi:10.1186/s13075-015-0669-3.
47. Khanna D, Spino C, Johnson S, Chung L, Whitfield ML, Denton CP, et al. Abatacept in Early Diffuse Cutaneous Systemic Sclerosis: Results of a Phase II Investigator-Initiated, Multicenter, Double-Blind, Randomized, Placebo-Controlled Trial. Arthritis Rheumatol. 2020;72(1):125-136. doi:10.1002/art.41055.
48. Distler O, Highland KB, Gahlemann M, Azuma A, Fischer A, Mayes MD, et al; SENSCIS Trial Investigators. Nintedanib for Systemic Sclerosis-Associated Interstitial Lung Disease. N Engl J Med. 2019;380(26):2518-2528. doi:10.1056/NEJMoa1903076.
49. Acharya N, Sharma SK, Mishra D, Dhooria S, Dhir V, Jain S. Efficacy and safety of pirfenidone in systemic sclerosis-related interstitial lung disease-a randomized controlled trial. Rheumatol Int. 2020;40(5):703-710. doi:10.1007/s00296-020-04565-w.
50. Khanna D, Denton CP, Jahreis A, van Laar JM, Frech TM, Anderson ME, et al. Safety and efficacy of subcutaneous tocilizumab in adults with systemic sclerosis (faSScinate): a phase 2, randomized, controlled trial. Lancet. 2016;387(10038):2630-2640. doi:10.1016/S0140-6736(16)00232-4.
51. Khanna D, Lin CJF, Furst DE, Goldin J, Kim G, Kuwana M, et al; focuSSced investigators. Tocilizumab in systemic sclerosis: a randomized, double-blind, placebo-controlled, phase 3 trial. Lancet Respir Med. 2020;8(10):963-974. doi:10.1016/S2213-2600(20)30318-0.
52. Matteson EL, Kelly C, Distler JHW, Hoffmann-Vold AM, Seibold JR, Mittoo S, et al. Nintedanib in Patients with Autoimmune Disease-Related Progressive Fibrosing Interstitial Lung Diseases: Subgroup Analysis of the INBUILD Trial. Arthritis Rheumatol. 2022;74(6):1039-1047. doi:10.1002/art.42075.
53. Vigone B, Caronni M, Severino A, Bellocchi C, Baldassarri AR, Fraquelli M, et al. Preliminary safety and efficacy profile of prucalopride in the treatment of systemic sclerosis (SSc)-related intestinal involvement: results from the open label cross-over PROGASS study. Arthritis Res Ther. 2017;19(1):145. doi:10.1186/s13075-017-1340-y.
54. Low AHL, Teng GG, Pettersson S, de Sessions PF, Ho EXP, Fan Q, et al. A double-blind randomized placebo-controlled trial of probiotics in systemic sclerosis associated gastrointestinal disease. Semin Arthritis Rheum. 2019;49(3):411-419. doi:10.1016/j.semarthrit.2019.05.006.
55. Marighela TF, Arismendi MI, Marvulle V, Brunialti MKC, Salomão R, Kayser C. Effect of probiotics on gastrointestinal symptoms and immune parameters in systemic sclerosis: a randomized placebo-controlled trial. Rheumatology (Oxford). 2019;58(11):1985-1990. doi:10.1093/rheumatology/kez160.
